# Morphological and immunohistochemical phenotype of TCs in the intestinal bulb of Grass carp and their potential role in intestinal immunity

**DOI:** 10.1038/s41598-020-70032-y

**Published:** 2020-08-20

**Authors:** Hanan H. Abd-Elhafeez, Alaa S. Abou-Elhamd, Soha A. Soliman

**Affiliations:** 1 Department of Anatomy, Embryology and Histology, Faculty of Veterinary Medicine, Assiut University, Assiut, 71526 Egypt; 2 Department of Histology, Faculty of Veterinary Medicine, South Valley University, Qena, 83523 Egypt

**Keywords:** Biological techniques, Cell biology, Structural biology

## Abstract

The current study investigated telocytes (TCs) in the intestinal bulb of Grass carp using light microscopy (LM), Transmission electron microscopy (TEM), scanning electron microscopy, and immunohistochemistry (IHC). By LM, TCs were distinguished by the typical morphological features that had a cell body and telopodes using HE, toluidine blue, methylene blue, Marsland silver stain, Grimelius’s silver nitrate, Giemsa, PAS, combined AB pH2,5/PAS, Crossmon’s and Mallory triple trichrome, Van Gieson stains, Verhoeff’s stain, Sudan black, osmic acid, performic acid with methylene blue and bromophenol blue. TCs were identified under the epithelium as an individual cell or formed a TCs sheath. They detected in the lamina propria, between muscle fibers, around the myenteric plexus and fibrous tissue. TCs acquired immunological features of endocrine cells that exhibited high affinity for silver stain, performic acid with methylene blue, Marsland stain, and immunohistochemical staining using chromogranin A. Sub epithelial TCs were closely related to the endocrine cells. TCs and their secretory activities were recognized using acridine orange. TCs were identified by IHC using CD34, CD117, S100-protein, desmin. TCs formed a3D network that established contact with macrophage, mast cells, dendritic cells, lymphocytes, smooth muscle fibers, fibroblast, Schwann cells and nerve fibers. In conclusion, the localization of TCs in relation to different types of immune cells indicated their potential role in the maintenance of intestinal immunity.

## Introduction

Interstitial cells have critical roles in the maintenance of the appropriate 3D scaffold and functional requirements of the organs. Telocytes (TCs) are the cell population that forms a network through a labyrinthine system formed by telopodes. They are long and slender prolongations reached up to hundreds of microns. Telopodes are identified by their segments; the thin segment, podomer and the thick segment, the podoms. TCs established contact to other resident cells or to wandering cells via their telopodes or via the cell body. Telopodes network provide—long-distance cell–cell signaling (intercellular communication)^1^.

Paracrine signaling is critical for TCS function. TCs produce several secretory molecules and factors contributing to the functional significance.Gene analysis^2^ and proteomics analysis^3^ are investigated for TCs. The functional contributions of TCS are supposed to be related to angiogenesis^4^, and development, maintenance of homeostatic balance^4^, immunosurveillance^5^, tissue regeneration and repair through providing adequate microenvironment for stem cell niche and promoting their differentiation^6,7^.

TCs are identified in a wide variety of organs from diverse species including mammals^8–11^, avian^12,13^, reptiles^14^, Amphibians^15^ and aquatic species^16,17^ and parasitic worm^18^. They are located in the trachea and lungs^19^, heart^20^ and the blood vessels^21^, kidney, ureter, urinary bladder^22^, tongue^23^, oesophagus, stomach and small and large intestines^24^, liver , pancreas^14^, testis^25^, prostate^26^, efferent ductules^27^ ovaries, oviducts, uterus, vagina, mammary glands and placenta^26^, spleen^8,28^.

TCs immunoprofile is recognized in several species. CD34/PDGFRα is specific markers for TCs^29^. TCs express other markers such as desmin, vimentin, SMA, tubulin, VEGF, and others^8,27^. The current study investigates the distribution, morphology, immunohistochemical characterization of TCs in the intestinal bulb of Grass carp.

## Material and methods

### Sample collection

The present work was carried out on eight specimens of adult grass carp or white amur (*Ctenopharyngodon idella*) (Order: Cypriniformes, Family: Cyprinidae) with the average mean standard length was 36.40 ± 3.01 cm, and the mean standard body mass was 506.40 ± 9.60 g.

All fish were anaesthetized using benzocaine (4 mg/L) and decapitated. The gastrointestinal tract was carefully excised after abdominal incision. The intestinal bulb, an anterior intestinal dilatation, was obtained and incised to wash and remove the mucous coat of the mucosal surface.

Four fish were taken for light microscopic examination, two fish for Scanning electron micrograph, and two fish transmission electron microscopic examination.

### Sample fixation and processing

#### Light microscopic examination

##### Fixation and processing of samples of paraffin-embedded samples for conventional and histochemical staining

Samples from the intestinal bulb were fixed in Bouin’s solution (Table 1). Samples were processed according to the description by^30^ as the following: The fixed samples thoroughly washed in 70% ethanol (3 × 24 h) to get rid of the fixative before subsequent processing. The samples were dehydrated in ascending grades of alcohol (80%, 90%, 100I, 100% II), cleared in methyl benzoate, and then embedded in paraffin wax for three hrs. Serial transverse and longitudinal sections of 5–7 µm were prepared by using a Richert Leica RM 2125 Microtome, Germany. For general histological examination, paraffin representative sections were stained by Hematoxylin and Eosin satin^31^.

**Table 1:** Components of the fixative.

Fixative	Components	Amount
Bouin’s solution	Parafomaldehyde 40%	25 mL
	Saturated picric acid	75 mL
	Glacial Acetic acid	5 mL
Karnovsky fixative	Paraformaldehyde, 25% freshly prepared	10 mL
	Glutaraldehyde 50%	10 mL
	Na-Phosphate buffer (0.1 M, pH 7.4)	50 mL
	Distilled water	30 mL
N a-Phosphate buffer (0.1 M, pH 7.4)	Solution A	
	Na_2_HPO_4_. _2_H_2_O Disodium hydrogen phosphate	17.02 gm
	Distilled water	600 mL
	Solution B	
	NaH_2_PO_4_ 2H_2_OSodium dihydrogen phosphate	6 gm
	Distilled water	200 mL
	Using solution	
	Solution A	580 mL
	Solution B	219 mL
Citrate-buffer (pH 6.0)	Solution A	
	Citric acid monohydrate C_6_H_8_O_7_.H_2_O	21 g
	Distilled water	1 L
	Solution B	
	Sodium citrate Na_3_C_6_H_5_O_7_. 2 H_2_O	29.41 g
	Distilled water	1 L
	Using solution	
	Solution A	9 mL
	Solution B	41 mL
	Distilled water	Add 500 mL

##### Histochemical investigation

The following histochemical stain used was cited in^32^: trichrome according to Crossmon’s^33^ and Mallory triple trichrome stain^34^, PAS^35^, Combined Alcian Blue pH 2.5 Periodic acid Schiff (AB pH 2.5 /PAS) technique^36^, Wiegert^37^, Verhoeff’s counter stain by Van Gieson^38^, Long ziehl neelsen, Giemsa stain, Grimelius’s silver nitrate^39^ and Marsland. Gless and Erikson method^40^.

All staining methods were cited by^32^

Leitz Dialux 20 Microscope provided with a Canon digital camera (Canon Power shot A95) used to examine the stained sections and capture the images.

##### Osmium tetroxide paraffin procedure for fat

After fixation of a small piece about 0.0.5 × 0.5 × 0.5 cm sample in 10% Neutral buffer formalin, they placed in osmium tetroxide for demonstration of fat by the method that allows paraffin embedded in the tissue [Connective and Muscle Tissue^41–43^] .

##### Acridine orange (fluorescent stain)

The procedure was performed according to that of Hoff et al. modified by Refs.^27,44–48^. Acridine Orange is a cationic dye and stains proteins-containing membranous vesicles including secretory vesicles, membrane bounded acidic compartments, and lysosomes that had an acidic nature. Acridine Orange exhibits a metachromatic reaction that associated with the liberation of green and red fluorescence. Acridine Orange reacts with the membrane bounded vesicles and appeared orange or red. Acridine Orange is used for the identification of secretory vesicles and lysosomes^49–51^.

The stained sections analyzed using a Leitz DM 2500 microscope with the external fluorescent unit Leica EL 6000.

##### Immunohistochemistry staining (IHC) for CD34, CD117

Immunohistochemically staining was performed on paraffin sections .Antigen localization was achieved using combined with the avidin–biotin complex (ABC) technique^52^. Using the Reagent of Ultra Vision Detection System (Anti-Polyvalent, HRP/DAB (ready to use, TP-015-HD: Thermo Fischer Scientific TP-015HD) according to the manufacturer’s instructions. The procedure according to a description of^53^ as the following:

Paraffin sections of (5 µm) were dewaxed by xylene, rehydrated by ascending grades of alcohols, and rinsed by PBS pH 7.4 (3 times for 5 min). Endogenous peroxidase was suppressed by using a hydrogen Peroxide block at room temperature. The sections were thoroughly washed by running tap water for an additional 10 min. To enhance antigen retrieval, the slides were treated with 10 mM sodium citrate buffer (Table 1) (pH 6.0) at a temperature reached 95–98 in a water bath for 20 min. The sections were cooled for 20 min from room temperature and subsequently were washed in PBS (pH 7.4, 3 times for 5 min). Block non-specific background staining was performed by using Ultra V block (Thermo Fisher scientific, UK. Lab Vision corporation; USA) for 5 min at room temperature. Ultra V block application did not exceed 10 min to avoid staining artifact). The sections were incubated with the primary antibodies (The used primary antibody, sources, dilutions, and time of incubation of each antibody are shown in (Table 2). Sections were washed using PBS (at pH 7.4, 3 times for 5 min). The Biotinylated secondary antibody was applied for one hour at room temperature. The (Table 2). Sectioned were washed in PBS (pH 7.4, 3 times for 5 min) and subsequently incubated with streptavidin- peroxidase complex ,Thermo Fisher Scientific, UK. Lab Vision corporation; USA) for10 min at room temperature. Visualization of the bound antibodies was performed using 1 drop of DAB plus chromogen to 2 mL of DAB plus substrate. The mixture was applied and incubated at room temperature for 5 min .The incubation processes were carried out in a humid chamber. Harris hematoxylin was used as counter stained for 30 s. The sections were dehydrated using ethanol and isopropanol I and II, cleared in xylene, and covered by DPX.

**Table 2:** Identity, sources, and working dilution of antibodies used in immunohistochemical studies. Antibodies used that showed reactivity in fish species in past publication.

Target	Primary antibody supplier	Origin (catalog no)	Dilution	Incubation	Antigen retrieval	Secondary antibody-incubation time
CD34	Rat Anti-mouse CD34 antibody ( e bioscience, San Diego, CA)	mouse CD34 Monoclonal Antibody (Clone: RAM34) (Cat.no 14–0.341-85)	1:100	Over night	Boiling in citrate buffer (pH 6.0), 20 min Goat	Biotinylated goat Anti-Polyvalent, Anti-mouse Igg + Anti-Rabbit Igg, Thermo Fisher Scientific, The UK. Lab Vision Corporation; USA One hour at room temperature
CD117 (Kit)	Anti-CD117 (e Bioscience, San Diego, CA)	Mouse Cd117 Monoclonal Antibody (Clone. ACK2) (Cat. no. 14–1172–82)	1:100	Over night	Boiling in citrate buffer (pH 6.0), 20 min	
S100 protein	Anti-S100 protein (Dako, Glostrup, Denmark;	Rabbit polyclonal Antibodycode No. Z0311	1:100	Overnight	Boiling in citrate buffer (pH 6.0), 20 min	Goat anti-rabbit secondary antibody (cat. no. K4003, Envision + TM System Horseradish Peroxidase Labelled Polymer; Dako) 30 min at room temperature
Chromogarnin A	Anti Chromogranin A-(Dako A0430)	Polyclonal, Rabbit anti-Human	1:1000	One hour at room temperature	boiling in citrate buffer (pH 6.0), 20 min	
Desmin	Thermo Fischer scientific (invitrogen)	Desmin recombinant rabbit monoclonal antibody Clone (sI18-00) Cat.MA5-32,068	1:50	One hourat room temperature	Boiling in citrate buffer (pH 6.0), 20 min	

Use a Leitz Dialux 20 Microscope provided by a cannon digital camera (Cannon Power shot A95) to examine the stained section.

##### Immunohistochemical procedures of Desmin, S100 protein, chromogranin A

A Technique using the reagent of The DAKO En Vision TM + System, HRP peroxidase. The DAKO Envision TM + System, HRP is a two-step immunohistochemical staining technique^54^.

The procedure of staining according to^55^ used the following protocol: sections (5 µm) of paraffin-embedded sections were dewaxed, rehydrated, and rinsed in PBS, pH 7.4 (3 times for 5 min). Endogenous peroxidase was inhibited by adding drops of 3% hydrogen peroxide in methanol at room temperature for 20 min followed by intense washing under running tap water for an additional 10 min. For antigen retrieval, slides were placed in 10 mM sodium citrate buffer (pH 6.0) (Table 1) and heated to 95–98 in a water bath for 20 min, followed by cooling for 20 min at room temperature. Sections were then rinsed in PBS (pH 7.4, 3 times × 5 min). Sections were covered by adding drops of blocking serum (Dako) to cover the sections for 5 min at room temperature to block non-specific background staining. (Note: Do not exceed 10 min or there may be a reduction in the desired stain.). Sections were then incubated with the primary antibody (Table 2: Identity, sources, and working dilution of antibodies used in immunohistochemical studies). After incubation, slides were washed with PBS (pH 7.4, 3 times × 5 min). Followed by incubation for 30 min at room temperature with secondary antibody at room temperature. The slides were thereafter rinsed in PBS (pH 7.4, 3 times for 5 min) followed by Incubation for 5–10 min at room temperature with 3,3′-diaminobenzidine (DAB) + substrate-chromogen which results in a brown-colored precipitate at the antigen site. The sections were counterstained with Harris Hematoxylin were used as a counters stained for 30 s. The sections were dehydrated using ethanol alcohol 90%, and 100% II, cleared in xylene, and covered by DPX and I Leitz Dialux 20 Microscope provided by a cannon digital camera (Canon Power shot A95) was used to examine immunohistochemical staining.

All Negative controls of the five markers were performed as the previous steps without adding the primary antibody.

#### Electron microscopic examination

A different small specimen about 3mm^3^ thickness was carefully excised and fixed in a Karnovsky fixative^56^ (Table 1) for preparation for scanning electron microscope and transmission electron microscope examination.

##### Preparations of resin embedding samples for semi thin and ultrathin sections

Abdel-Hafeez and Soliman^57^, Soliman, Ahmed et al.^58^, Soliman and Emeish^59^, Soliman^60^ described the procedure as follows: the samples were washed four times for 15 min in 0.1 M sodium phosphate buffer (pH 7.2) and then postfixed in 1% osmic acid in 0.1 M sodium phosphate buffer at 4 °C for 2 h. The samples were then washed again three times for 20 min in 0.1 M phosphate buffer (pH 7.2). Dehydration was performed by using ethanol gradient and propylene oxide. Samples were dehydrated in an ascending graded ethanol series [50% (for 30 min), 70% (overnight), 90% (for 30 min), 100% I (for 30 min), and 100% II (for 60 min]. The dehydrated samples were embedded in resin (Epon-Araldite as follows: propylene oxide (Merck, Darmstadt, Germany for 30 min, Epon: propylene oxide (approximately 1:1, for 30 min), followed by Epon (for 3 h). Epon was prepared as follows: 5 mL of Epon 812 (Polysciences, Eppelheim, Germany) + 5 mL of Araldite + 12 mL of DDSA. Then, the Epon was mixed thoroughly by incubation in a shaker at 60 °C. Sample polymerization was performed using Epon mix and an accelerator (DMP30) (1.5%). The blocks were incubated for 3 days as follows: 60 °C on the first day, 70 °C on the second day, and 75 °C on the third day.

Using an Ultra cut E (Reichert Leica RM2125, Microtome, Germany), 0.5–1 µm thick semi-thin sections were cut. The semi-thin sections were stained with toluidine blue^32^.

Ultrathin sections were obtained from semi-thin sections by a Reichert ultra-microtome. The sections (70 nm) were stained with uranyl acetate and lead citrate^61^ and examined by JEOL100CX II transmission electron microscope at the Electron Microscopy Unit of Assiut University.

##### Semi-thin sections stained by PAS stain

Additional resin-embedded specimens were used in PAS stain. Resin sections were treated with a saturated alcoholic solution of sodium hydroxide for 3 min to dissolve the resin^62^. Then processed the methods as usual PAS stain after immersion in ethanol alcohol 100%, 90%, 80%, 70% for 3 min in each concentration. The protocol used in the preparation of semi-thin sections was performed according to references^8,32^.

##### Digital coloring of scanning and transmission electron microscopic images

We applied digital coloring for the transmission electron microscopic images using the Photo Filter 6.3.2 program to identify different types of cells and structures. Many authors previously used the methods^12,63–67^.

##### CMEIAS color segmentation (for supplementary images)

Negative images were performed using CMEIAS Color Segmentation, an improved computing technology used to process color images by segmenting the foreground object of interest from the background^64^. This has been done by the following steps: open the image with CMEIAS Color Segmentation, then select “Process” from the menu items, and subsequently choose “Negative image”^68–71^.

### Ethical approval

The National ethics committee of Assiut University and veterinary authorities -from Assiut province, Egypt, were approved the method of work. "All methods were performed in accordance with the relevant guidelines and regulations".

## Results

**Figure 1:**
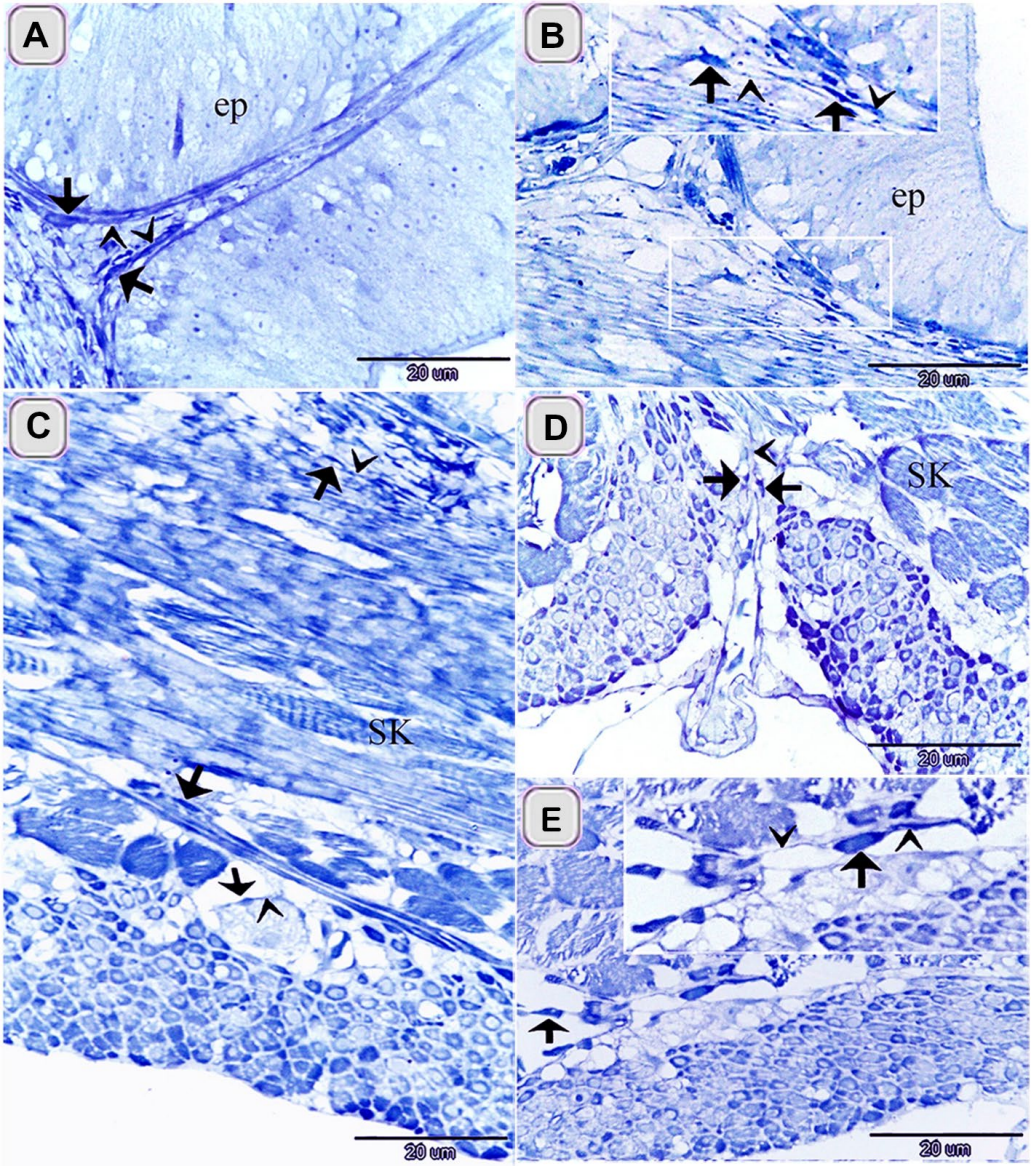
Identification of TCS in the intestinal bulb of Grass carp using methylene blue. Semi thin sectioned of the intestinal bulb stained by methylene blue. The cell body of TCs (arrows), telopodes (arrowheads) under the epithelium (**A**), in the lamina propria (**B**) and in between the muscular fibers (**C**–**E**). Note epithelium (ep), skeletal muscles (SK) in the initial part of intestinal bulb.

**Figure 2:**
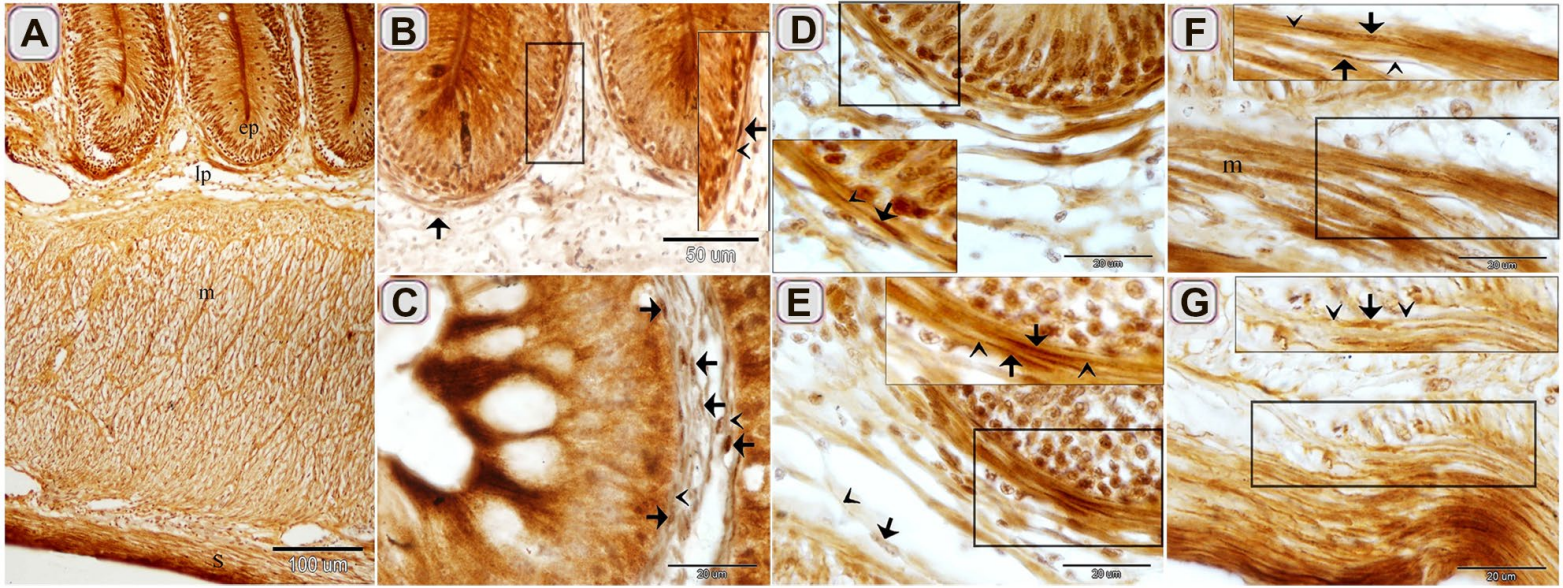
Identification of Telocytes in the intestinal bulb of Grass carp using Marsland silver stain. Paraffin sections of the intestinal blub stained by Marsland silver stain. (**A**) General histological picture of the intestinal bulb. Note epithlium (ep), lamina propria (lp), muscular layer (m), and serosa (s). (**B**, **C**, **E**) The cell body of TCs (arrows) formed a subepithelial sheath. Telopodes (arrowheads). (**D**) The cell body of the subepithelial TC (arrow). Telopodes (arrowheads). (**F**, **G**) The cell body of the intramuscular TC (arrow). Note muscle (m). Telopodes (arrowheads).

**Figure 3:**
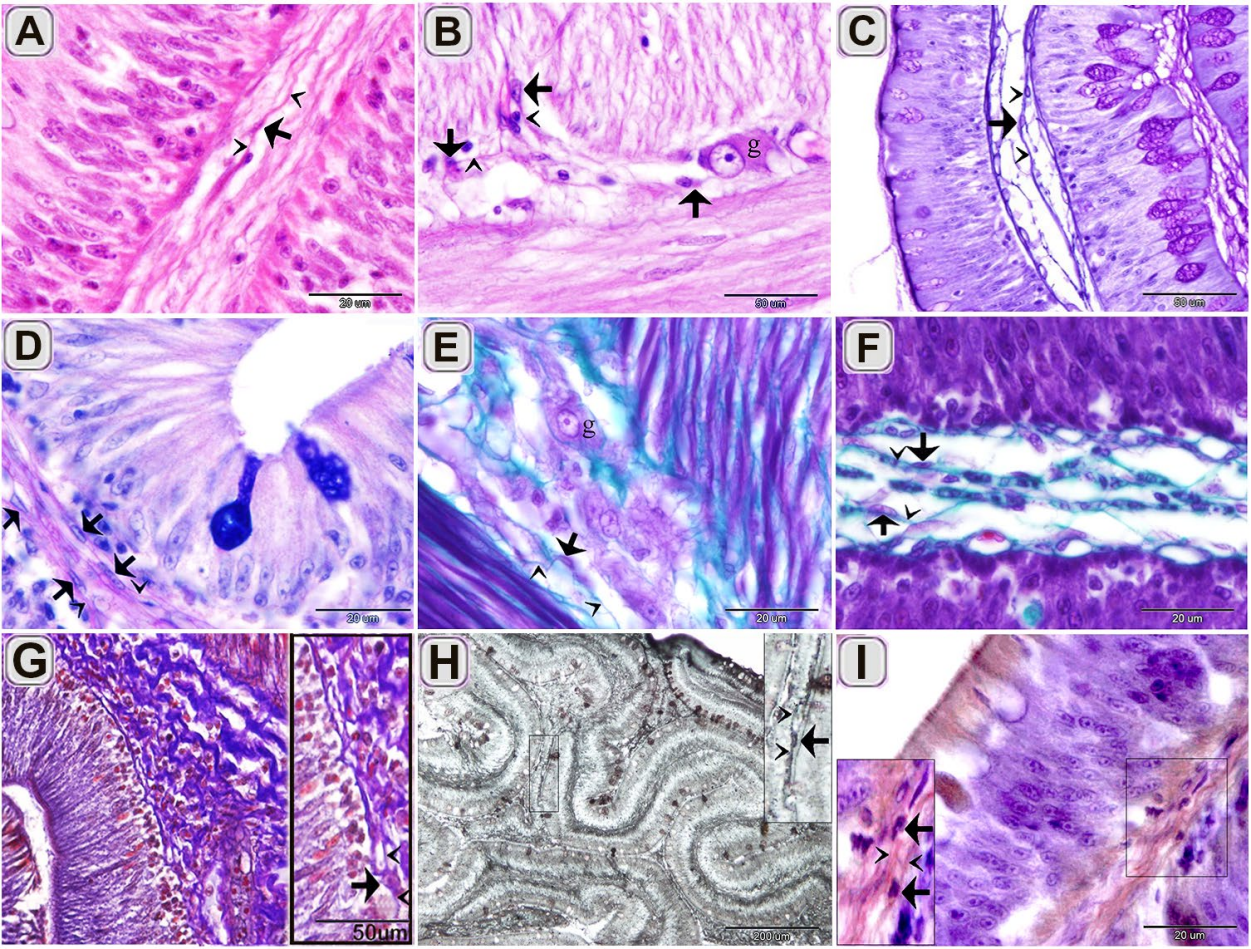
Recognition of TCs using HE, PAS, AB pH 2.5/PAS, Crossmon trichrome, Mallory triple trichrome , Sudan black, osmic acid. Paraffin sections of the intestinal blub stained by HE (**A**, **B**), PAS/Hx (**C**), AB ph 2.5/PAS (**D**), Crossmon trichrome (**E**, **F**), Mallory triple trichrome (**G**), Sudan black (**H**), osmic acid (**I**). (**A**) The cell body of TC (arrow) in the lamina propria was recognized by telopodes. Telopodes (arrowheads). (**B**) The cell body of TCs (arrows) around the ganglionic cell (g) of the myenteric plexus. Telopodes (arrowheads). (**C**) TC stained positively for PAS. Telopodes (arrowheads). (**D**) TC had a strong affinity for PAS rather than AB. Telopodes (arrowheads). (**E**) The cell body of TCs (arrows) stained green by Crossmon trichrome. Note the ganglionic cell (g) of the myenteric plexus. (**F**) TC (arrow) in the lamina propria stained green by crossmon trichrome. Telopodes (arrows). (**G**) The cell body of TCs (arrows) stained blue by Mallory triple. Telopodes (arrowheads). (**H**) The cell body of TC (arrow) stained black by Sudan black. Telopodes (arrowheads). (**I**) The cell body of TC (arrow) stained brown by osmic acid. Telopodes (arrowheads).

**Figure 4:**
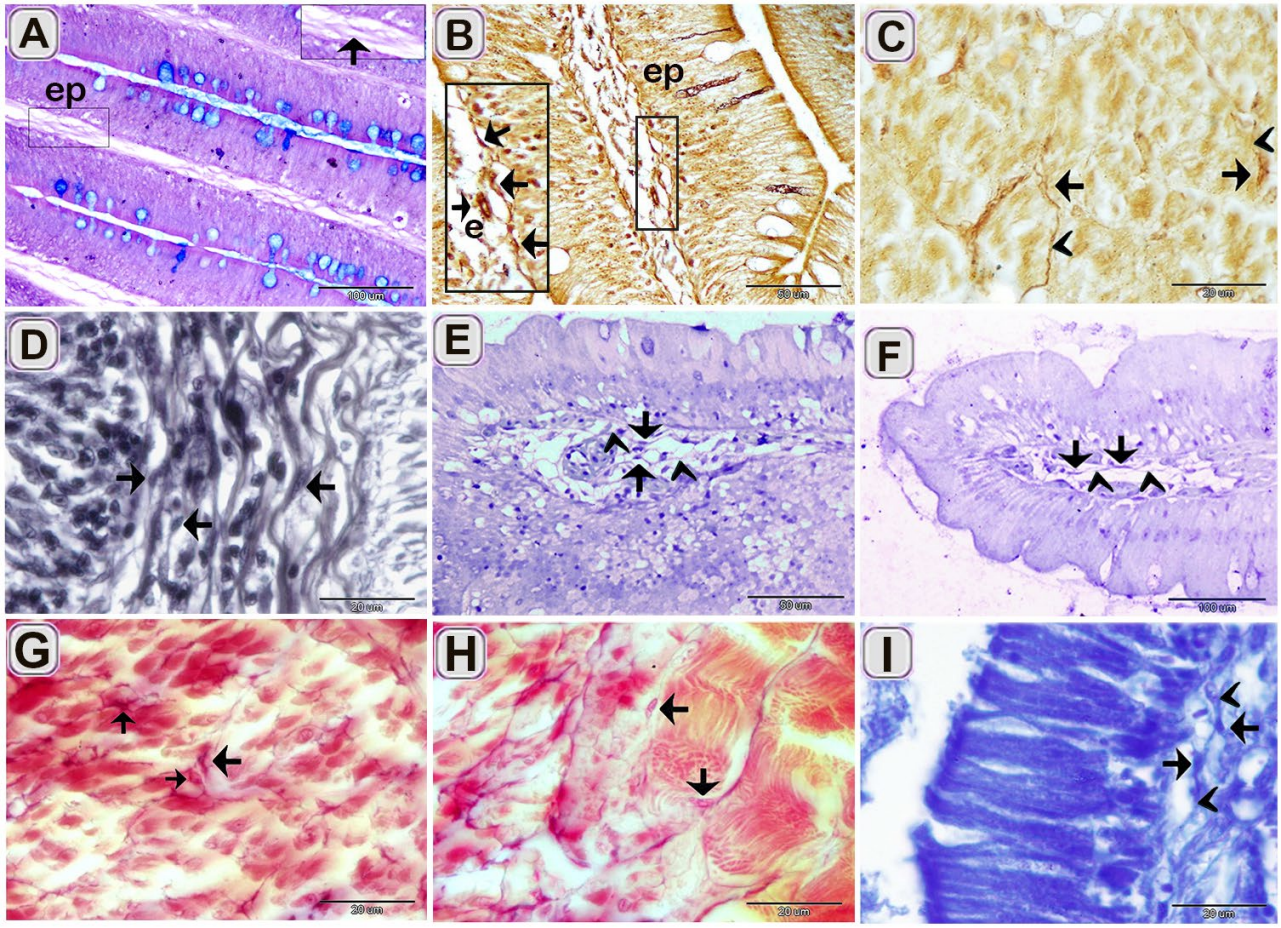
Recognition of TCs using Grimelius’s silver nitrate, Verhoeff stain, Giemsa, Weigert Van Gieson, bromophenol blue. Paraffin sections of the intestinal blub stained by Long ziehl neelsen (**A**), Grimelius’s silver nitrate (**B**, **C**), Verhoeff stain (**D**), Giemsa (**E**, **F**), Weigert van Gieson (**G**, **H**), bromophenol blue (**I**). (**A**) The cell body of subepithelial TC (arrow) stained bluish color that had affinity for Methylene blue. Note epithelium (ep). (**B**) The cell body of the subepithelial Telocytes (arrows) in closed vicinity to endocrine cell (e,arrow). Note epithelium (ep). (**C**) The cell body of TCs (arrows) located between muscles cells. Telopodes (arrowheads). (**D**) TC (arrow) stained black by Verhoeff stain. (**E**, **F**) TC (arrow) stained blue by Giemsa. Telopodes (arrowheads). (**G**, **H**) TC (arrow) stained positive for Van Gieson. Telopodes (arrowheads). (**I**) TC stained positive for bromophenol blue. Telopodes (arrowheads).

**Figure 5:**
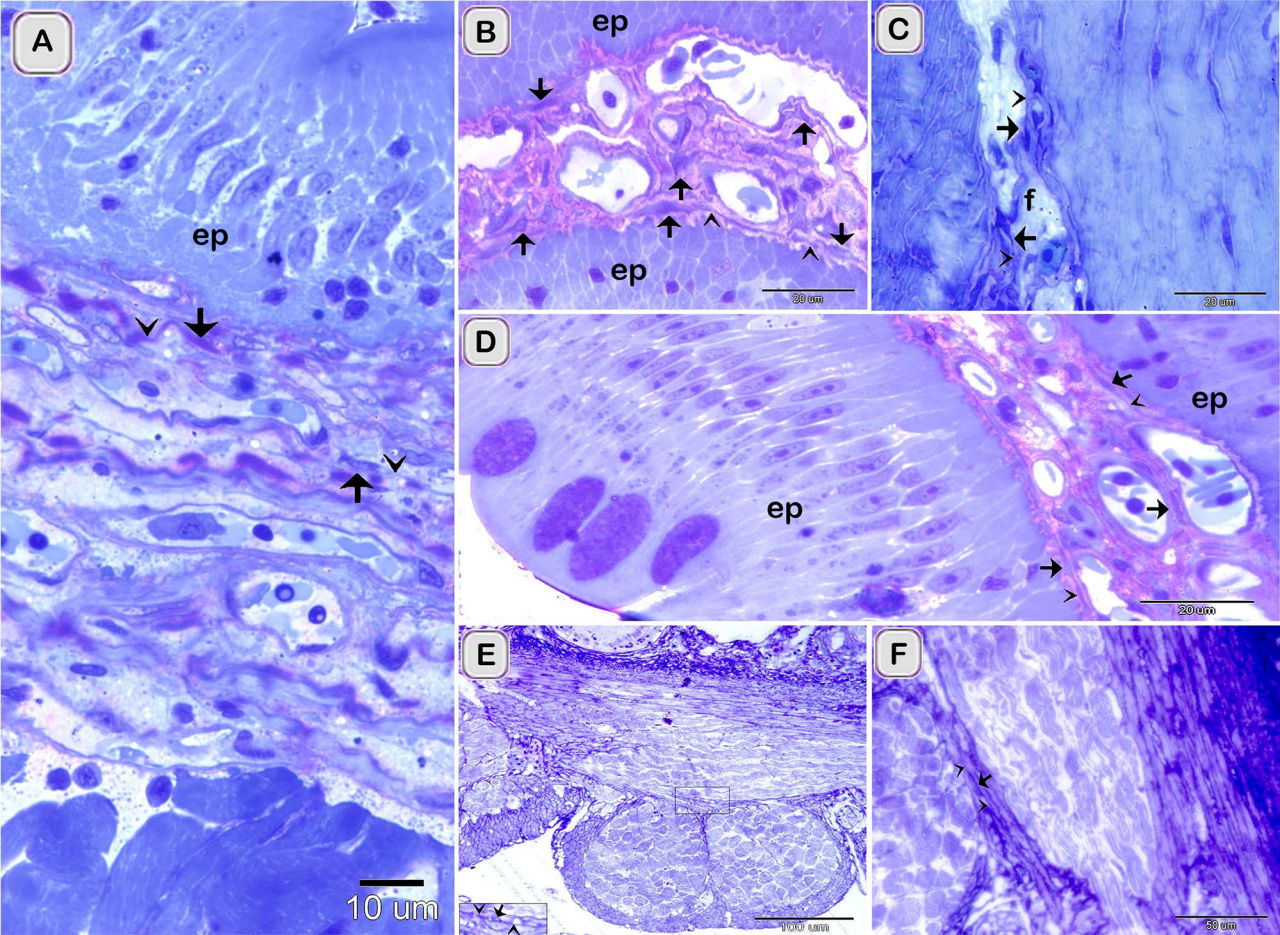
Recognition of TCs using semi thin sections. Semi thin sections stained by toluidine blue. (**A**, **B**, **D**) The cell body of TCs (arrows) under epithelium (ep) and in the lamina propria. Telopodes (arrowheads). (**C**) The cell body of TCs (arrows) in the fibrous tissue (f). Telopodes (arrowheads). (**E**, **F**) The cell body of TCs (arrow) located between the muscle fibers and bundles. Telopodes (arrowheads).

**Figure 6:**
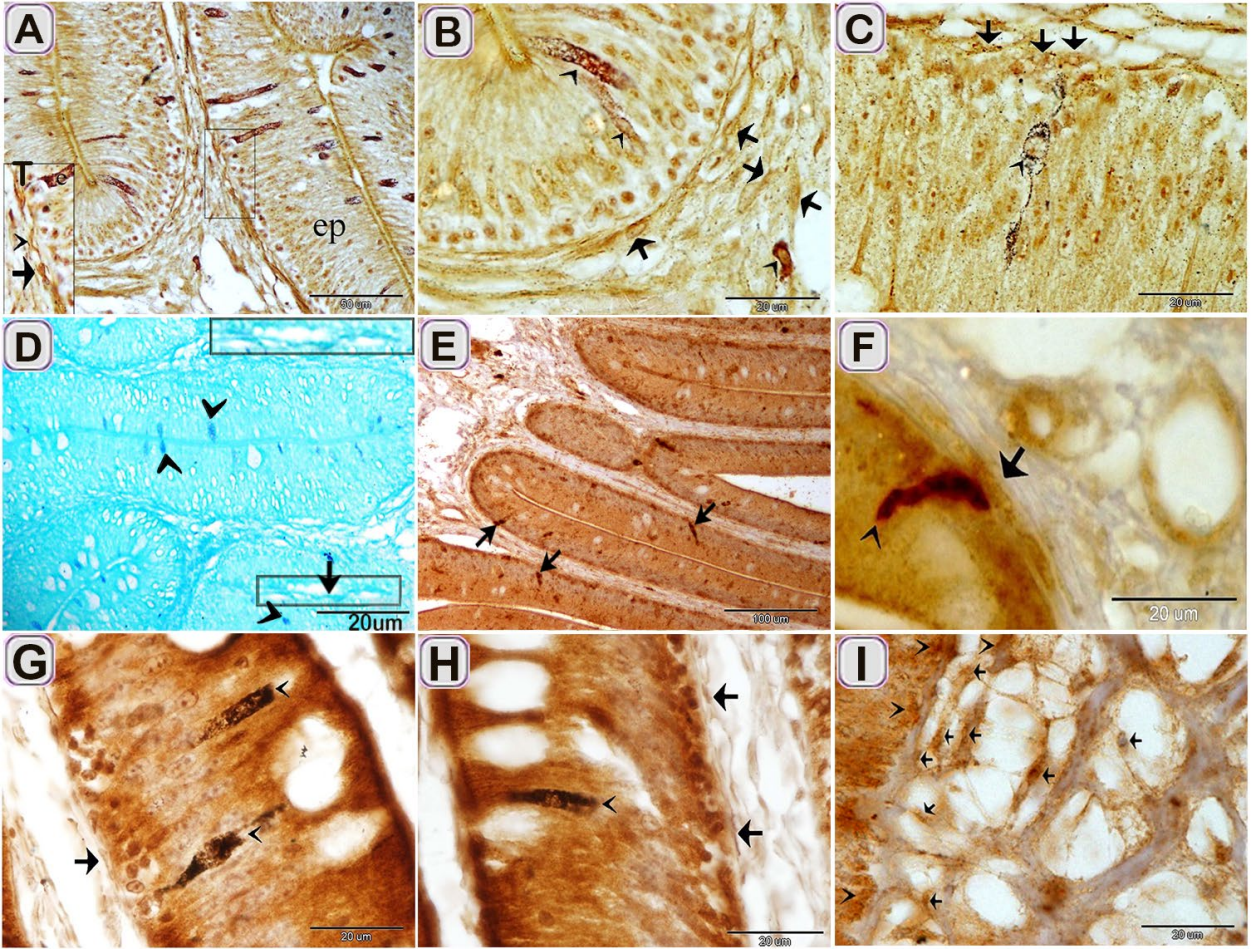
Relation between TCs and epithelial and interstitial endocrine cells using Grimelius’s silver nitrate stain, performic acid with methylene blue, Marsland stain, and immunohistochemical staining using chromogranin A. Paraffin sections stained by Grimelius’s silver nitrate stain, (**A**–**C**), performic acid with alcian blue (**D**), Marsland stain (**G**, **H**), chromogranin A (**E**, **F**, **I**). (**A**–**C**) subepithelial TCs (T, arrows) were closely related to endocrine cells (arrowheads). (**D**) subepithelial TCs (arrows) were closely related to endocrine cells (arrowheads). (**E**) chromogranin positive cells within the lining epithelium (arrows). (**F**, **G**, **H**, **I**) subepithelial and interstitial TCs (arrows) were closely related to endocrine cells (arrowheads).

The current study investigated TCs in the intestinal bulb of the Grass carp. The intestinal bulb consisted of epithelium, lamina propria, muscular layer, serosa (Fig. 2A). Light microscope (LM), Transmission electron microscope (TEM), Scanning electron microscope (SEM), and immunohistochemistry (IHC) recognized Telocytes (TCs). For light microscopic examination, their typical morphological features distinguished TCs that had a cell body contained the nucleus and telopodes. TCs recognized by H&E (Fig. 3A,B) and toluidine blue blue (Fig. 5A–F). They had high affinity for nervous tissue-specific staining; methylene blue (Fig. 1A–E), Marsland silver stain (Fig. 2A–G), Grimelius’s silver nitrate impregnation (Fig. 4B,C) and Giemsa (Fig. 4E,F). TCs had strong affinity for PAS (Fig. 3C), combined AB ph2.5 /PAS stain (Fig. 3D), took the affinity of PAS only. They had a strong affinity for collagen fiber-specific stains including Light green (Fig. 3E,F), Methyl blue (Fig. 3G). While Tc tend to stain by Verhoeff stain (Fig. 4D) and exhibited a reaction similar to elastic fibers rather than collagen fibers when stained by Combined Weigert counter by Van Gieson stain and Van Gieson (Fig. 4G,H). They were visible in paraffin sections stained by Sudan black (Fig. 3H) and osmic acid (Fig. 3I). They stained for protein-specific stain performic acid with Methylene blue and bromophenol blue (Figs. 4I, 6C)

TCs were located under the epithelium as an individual cell (Figs. 1A,2D,3G, H, 4A, B,5A–C) or formed a TCs sheath (Figs. 2 B, C, E, 3D), lamina propria (Figs. 1B,3A, C, F, I,4D–F, I, 5A–C), between muscle fibers (Figs. 1,2F, G, 4C, G, H), between muscle bundles (Figs. 1C–E, 5 E, F), around the myenteric plexus (Fig. 3B, E) and fibrous tissue (Fig. 5C).

TCs acquired immunological features of endocrine cells. Relation between TCs and epithelial and interstitial endocrine cells was recognized using Grimelius’s silver nitrate stain (Fig. 6A–c)., performic acid with methylene blue((Fig. 6D), Marsland stain (Fig. 6G,H) and Immunohistochemical staining using chromogranin A (Fig. 6E,F,I).Chromogranin A positive TCs in the lamina propria (Fig. 6F,I). The Negative images were represented as a supplementary figure (supplementary Fig. 6).

**Figure 7:**
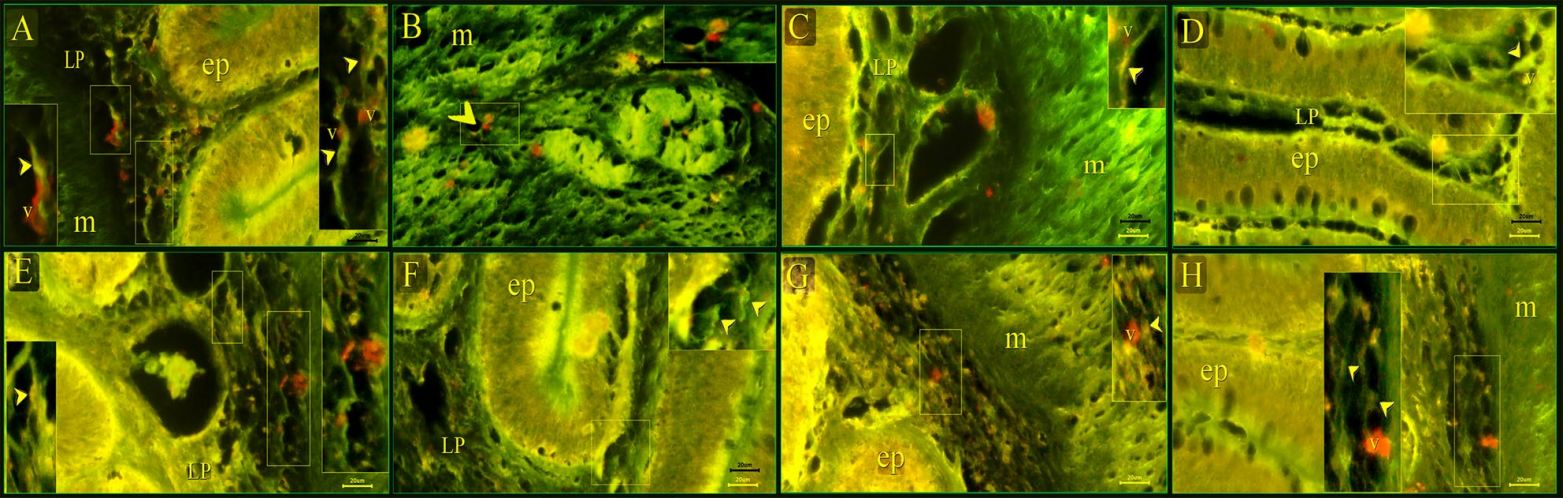
Recognition of TCs using Acridine orange. (**A**, **C**, **E**, **G**, **H**) TC in the lamina propria (arrowhead). Note secretory vesicle (v) associated with TC. (**B**) intramuscular TC (arrowhead). Note the secretory vesicle (v) associated with TC. (**D**, **F**) subepithelial TC (arrowhead). Note secretory vesicle (v) associated with TC.

TCs and their secretory activities were recognized using Acridine orange. They were distributed under the epithelium in the (Fig. 7D,F), in the lamina propria (Fig. 7A,C,E,G,H), in the muscular layer (Fig. 7B).

**Figure 8:**
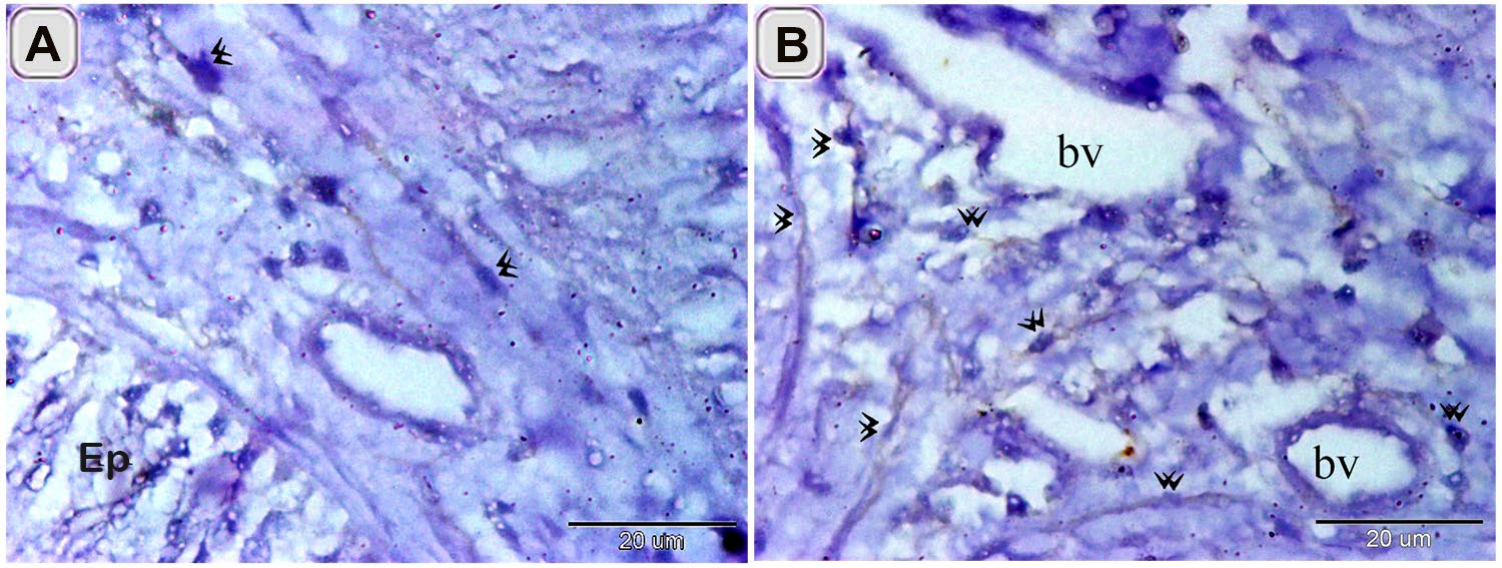
Immunohistochemical staining of the intestinal blub using CD34. Immunostained paraffin sections of the intestinal blub for CD34. counter stain by HX A: CD34 TCs (double arrowheads) in the subepithelial layer (Ep). B: CD34 TCs (double arrowheads) around the blood vessels (bv).

Localization of TCs in the intestinal blub of Grass carp was confirmed by Immunohistochemical staining. CD34 positive TCs were detected in the sub epithelial layer (Fig. 8A), around the blood vessels in the lamina propria (Fig. 8B). The Negative images were represented as a supplementary figure (supplementary Fig. 1).

**Figure 9:**
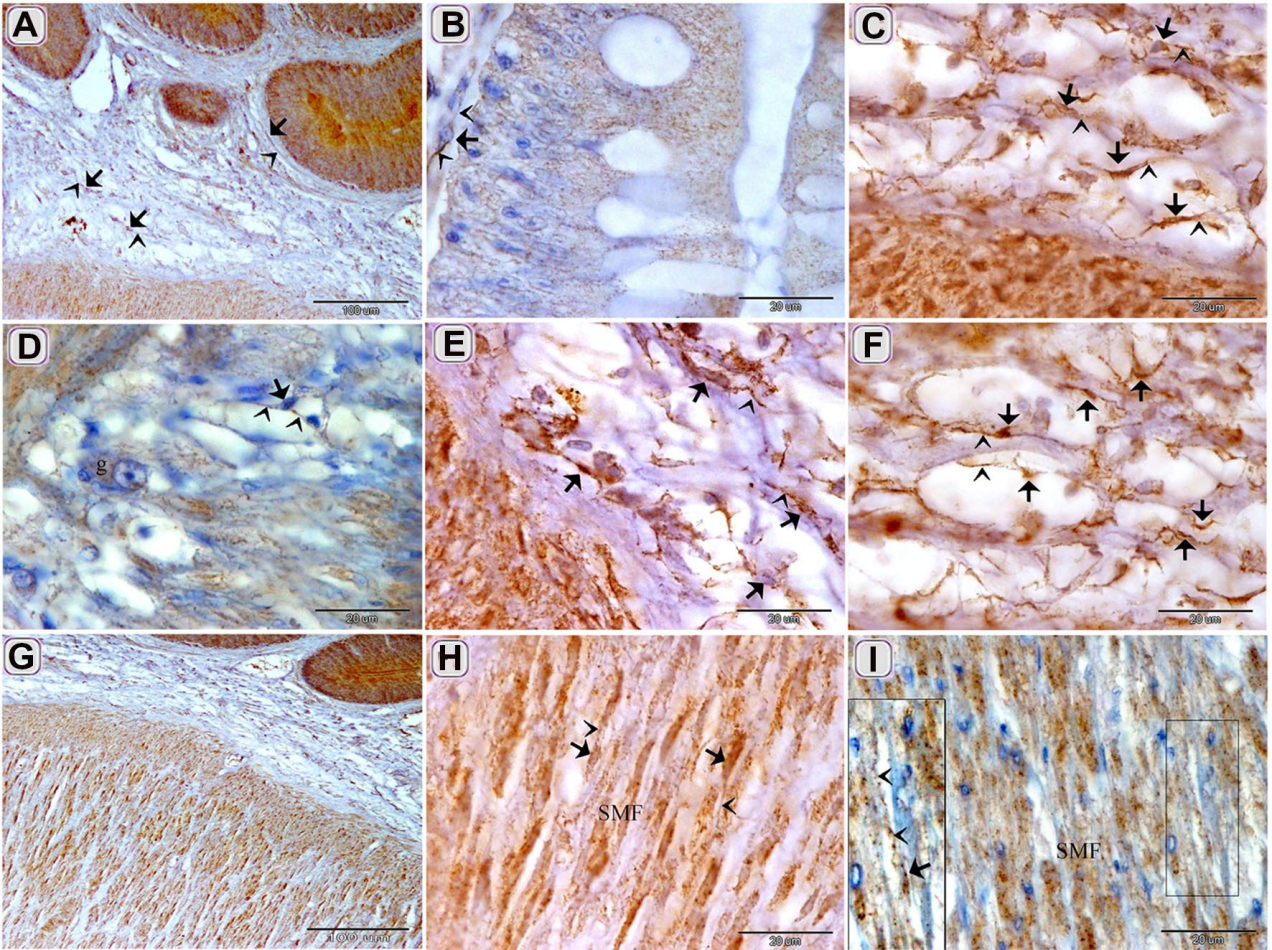
Immunohistochemical staining of the intestinal blub using CD117 counterstain by Hx except figure H. Immunostained paraffin sections of the intestinal blub for CD117. (**A**) CD117 positive TCs (arrows) under the epithelium and in the lamina propria. (**B**) CD117 positive sub epithelial TC (arrows). (**C**, **E**, **F**) CD117 positive TCs (arrows) in the lamina propria. (**D**) CD117 positive TC (arrows) around the ganglionic cell (g) of the myenteric plexus. (**G**, **H I**) CD117 positive TC (arrows) within the muscle layers. Note: Telopodes (arrowheads) and smooth muscle fiber (SMF).

CD117 positive TCs were detected under the epithelium (Fig. 9A,B), in the lamina propria (Fig. 9A,C,E,F), between MSF (Fig. 9G–I) and around the myenteric plexus. (Fig. 9D). The Negative images were represented as a supplementary figure (supplementary Fig. 2).

**Figure 10:**
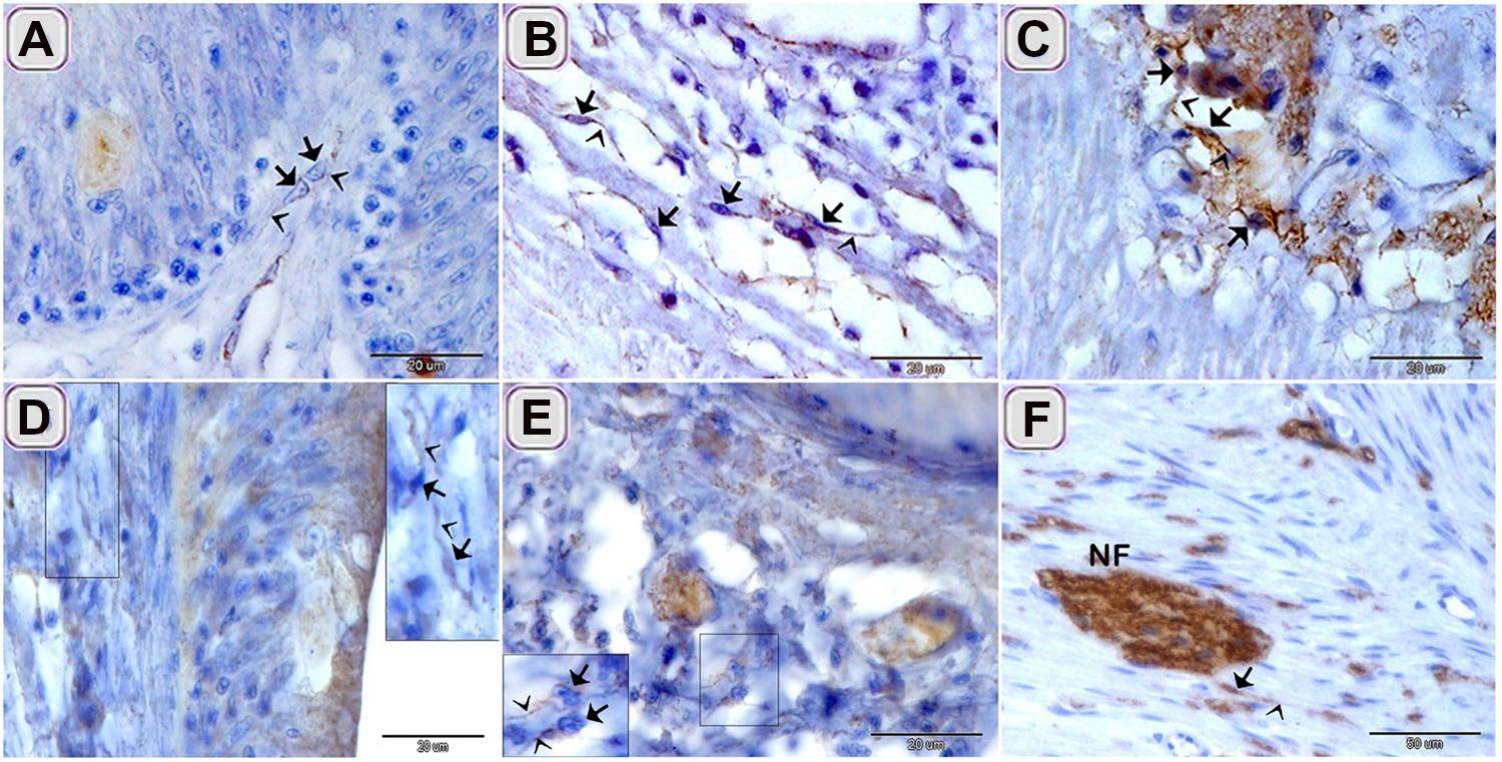
Immunohistochemical staining of the intestinal blub using desmin and S100-protein counter stain by HX. Immunostained paraffin sections of the intestinal blub for desmin (**A**–**C**) and S100-protein (**D**–**F**). (**A**) desmin positive sub-epithelial TCS (arrows). (**B**) desmin positive TCs (arrows) in the lamina propria. (**C**) desmin positive TCs (arrows) between the muscle bundles. (**D**) S100-protein positive TCs (arrows) under the epithelium. (**E**) S100-protein positive TC (arrows) between the muscle bundles. (**F**) S100-protein positive TC (arrows) connected with nerve fiber (NF). Note: Telopodes (arrowheads).

TCs exhibited immunohistochemical staining affinity for S100-protein in the lamina propria (Fig. 10D), between the muscle bundles (Fig. 10E), between the muscle fibers (Fig. 10F). Desmin positive TCs were identified in under the epithelium (Fig. 10A), in the lamina propria (Fig. 10B), and between the muscle bundles (Fig. 10C). The Negative images were represented as a supplementary figure (supplementary Fig. 3).

Chromogranin A positive TCs were detected in the lamina propria (Fig. 6E,F,I). The Negative images were represented as a supplementary figure (supplementary Fig. 6).

The images of the negative control were represented as a supplementary figure (supplementary Figs. 4, 5, 7 and 8).

**Figure 11:**
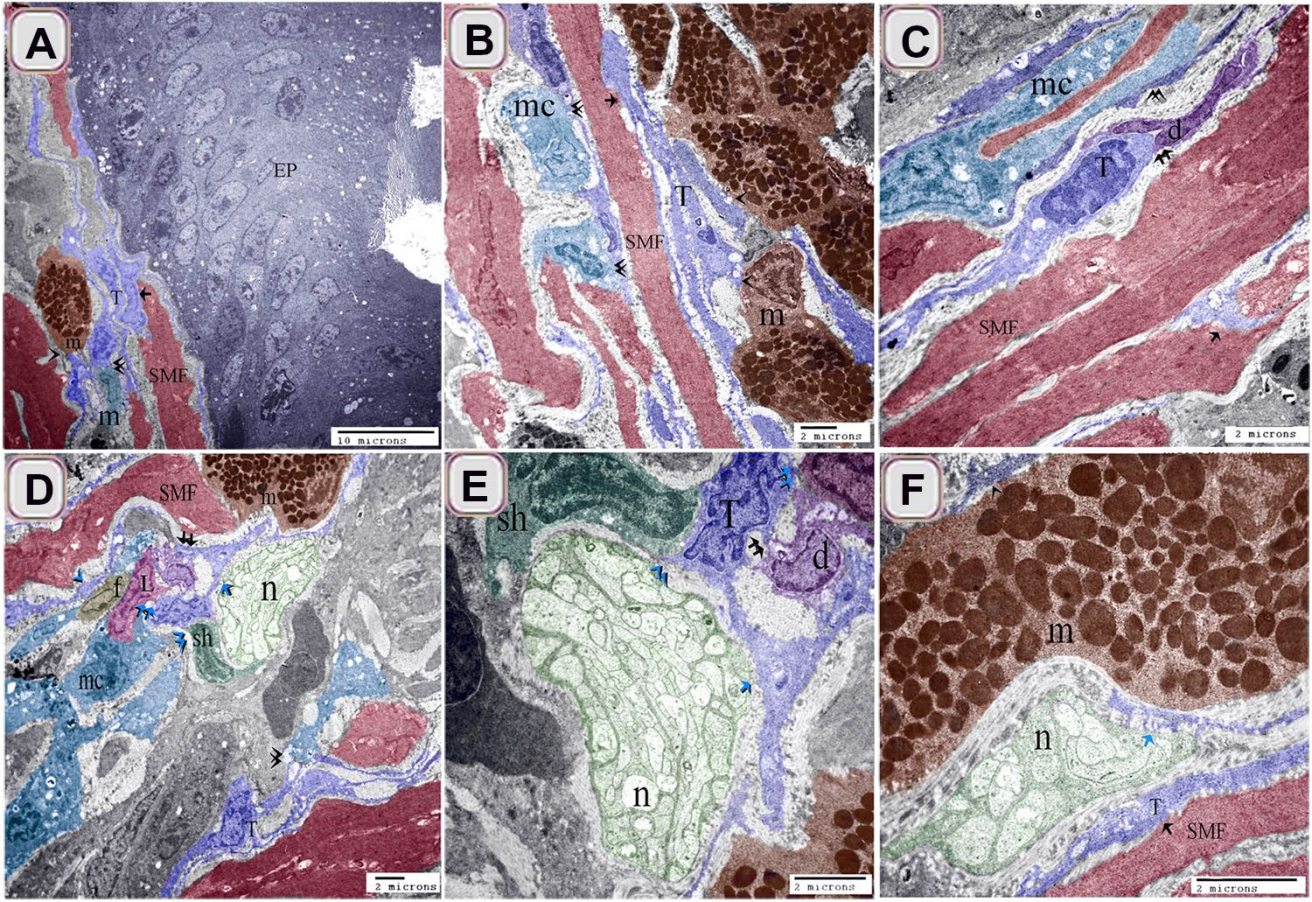
Relation of TCs with immune cells. (**A**) sub-epithelial TCS (T, blue color) and (**B**) TCS (T, blue color) in the lamina propria established a 3D network that connected with macrophages, (mc, turquoise color, double arrowhead), mast cells (m, brown color, arrowhead), and smooth muscle fibers (SMF, reddish brown, arrow). Note: epithelium (EP, gray color). (**C**) TCS (T, blue color) in the lamina propria connected with dendritic cells (d, violet color), macrophage (mc, double arrowhead), and smooth muscle fibers (SMF, reddish-brown) .(**D**–**F**) Tcs (T, blue color) connected with Schwann cell (sh, dark green color, blue double black arrowheads), nerve fibers (n, blue arrow), lymphocyte (L. blue double arrow), and dendritic cells (violet color, double arrow). Note mast cells (brown color, m), smooth muscle fibers (reddish-brown color, SMF), fibroblast (f).

**Figure 12:**
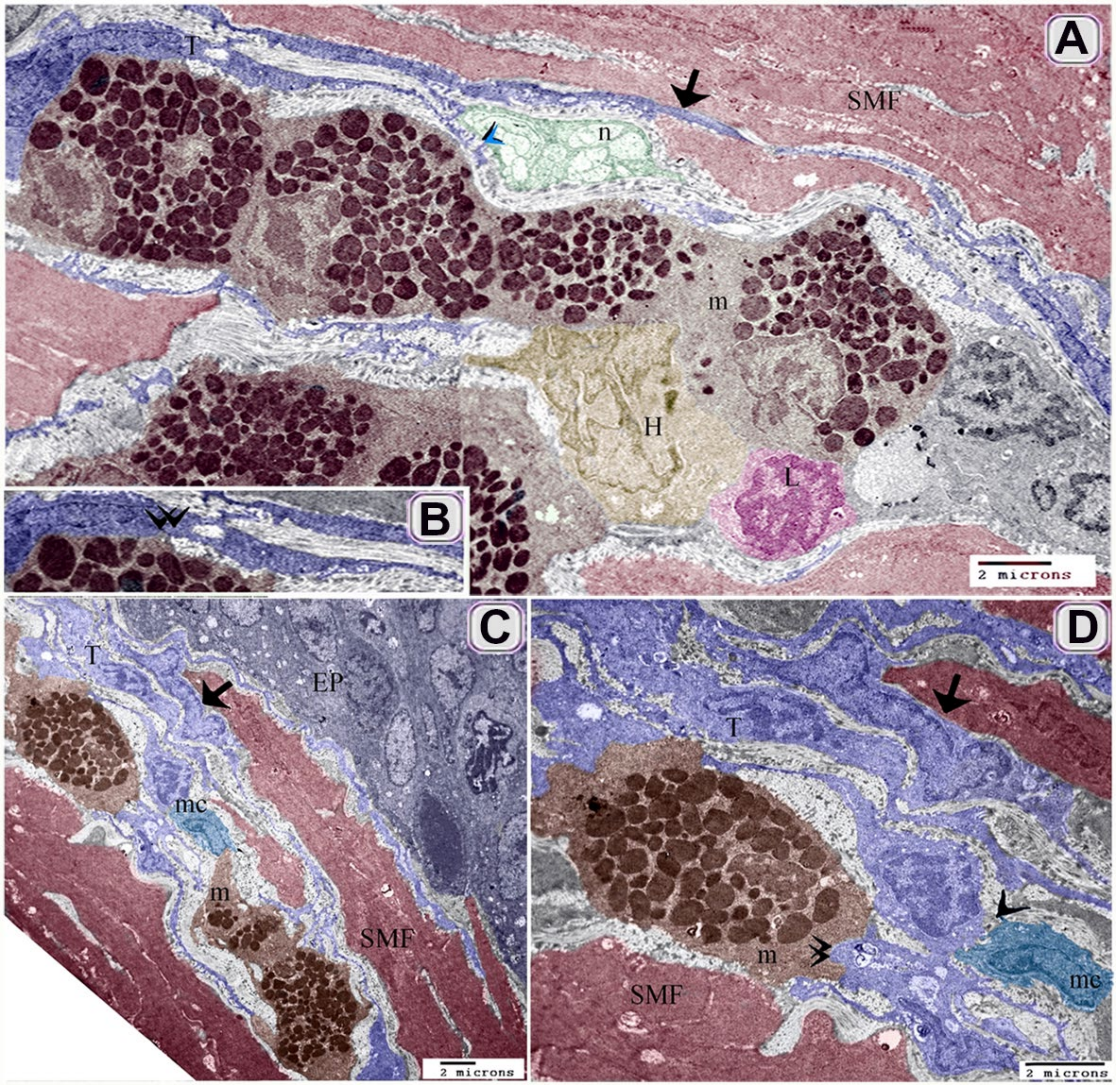
Relation of TCs with immune cells TEM. (**A**, **B**) TCS (T, blue color) connected with mast cells (m, brown color, and arrowhead), smooth muscle fibers (SMF, reddish-brown, arrow), nerve fibers (n, green color, blue arrow). Note heterophil (H, light brown), and lymphocyte (L, pink color). (**C**, **D**) sub epithelial TCS (T) established a 3D network that connected with macrophages (mc, the arrowhead), mast cells (m, brown color, double arrowhead), and smooth muscle fibers (SMF, reddish-brown color, arrow). Note epithelium (EP).

TCs established a 3D network that connected with different types of immune cells including macrophages (Figs. 11A–D, 12C,D), mast cells (Figs. 11A,B,D,F, 12A–D), dendritic cells (Fig. 11C–E), lymphocyte (Fig. 11D,E). They also formed contact with smooth muscle fibers (Figs. 11A–C, F, 12A,C,D), fibroblast (Fig. 11D), Schwann cells (Fig. 11D,E) and nerve fibers (Figs. 11 D–F, 12A).

By Transmission electron microscope (TEM), TCs connected with mast cells, smooth muscle fibers, nerve fibers (Fig. 12A,B). Subepithelial TCs established a 3D network that connected with macrophages, mast cells, and smooth muscle fibers (Fig. 12C,D).

**Figure 13:**
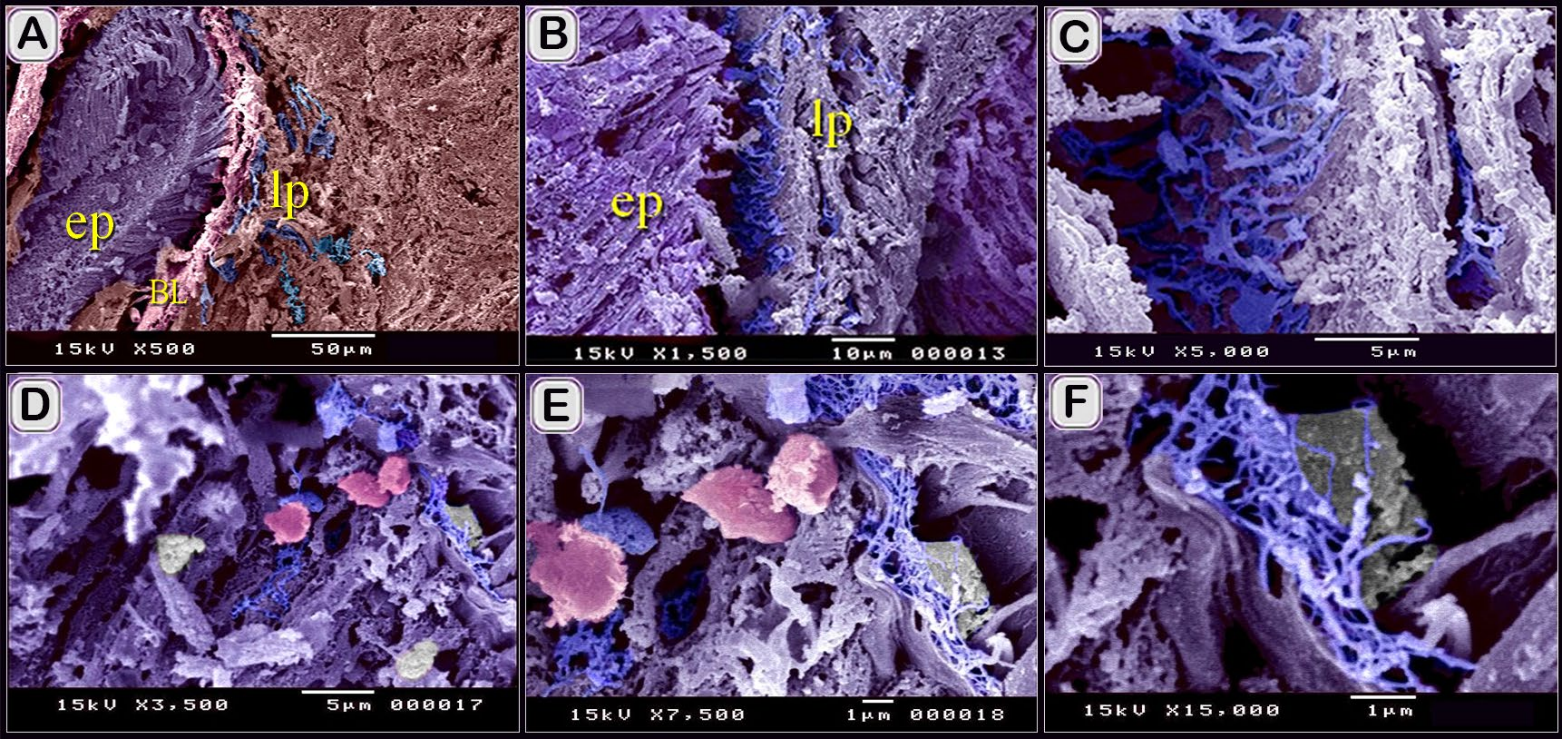
Scanned mucosal TC. (**A**) general view of the scanned sample of the intestinal blub. Note the epithelium (ep, violet color), lamina propria (lp), muscle (brown color). Basal lamina (Bl, pink color), TCs (blue colored). (**B**, **C**) sub epithelial TC sheet (blue colored). Note the epithelium (ep, violet color), lamina propria (lp). (**D**–**F**) TCs (arrowheads) in the lamina propria make contact with rodlet cells (pink colored) within the epithelium, were bear-shaped, and the capsules were smooth.

**Figure 14:**
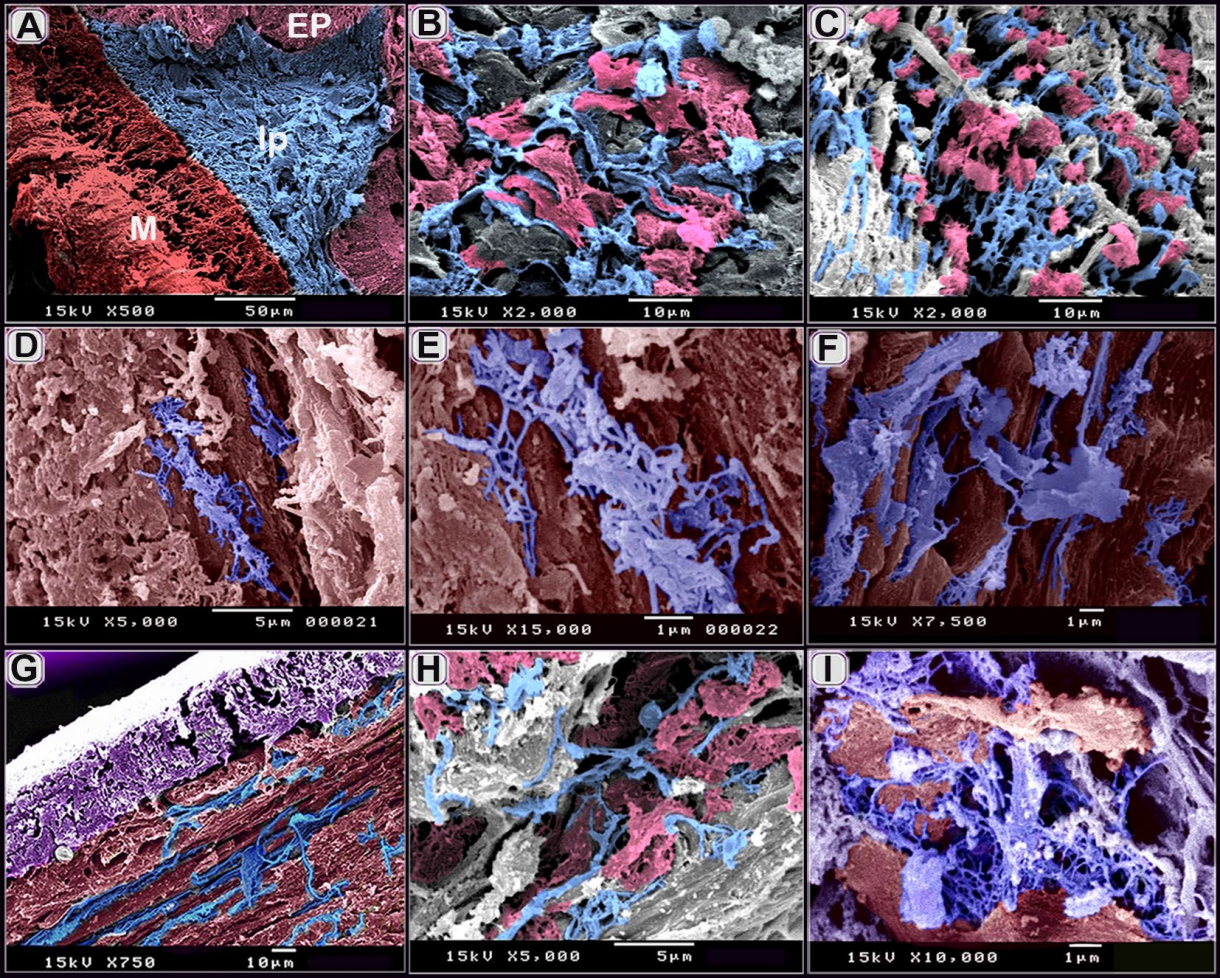
Scanned muscular TCs. (**A**) scanned sample of the intestinal blub. Note the epithelium (ep), lamina propria (lp, telocytes (blue color), muscle (m, reddish brown color). (**B**, **C**) TCs network (blue colored) in the lamina propria. Note connected teloped (pink colored). (**D**–**F**) TCs (blue colored) between muscle bundles (red colored). (**G**, **I**) TC network between muscle fibers (brown colored). Note connetced teloped (pink colored). (**H**) TCs (blue color) in serosa.

By Scanning electron microscope (SEM), Sub epithelial TC may form a continuous sheet (Fig. 13B,C). TCs were distributed in the lamina propria (Fig. 13A) where they connected to rodlet cells (Fig. 13D–F). TCs formed a network in the lamina propria (Fig. 14A–C). They were also identified between muscle bundles (Fig. 14D–G, I) and in the serosa (Fig. 14H).

**Figure 15:**
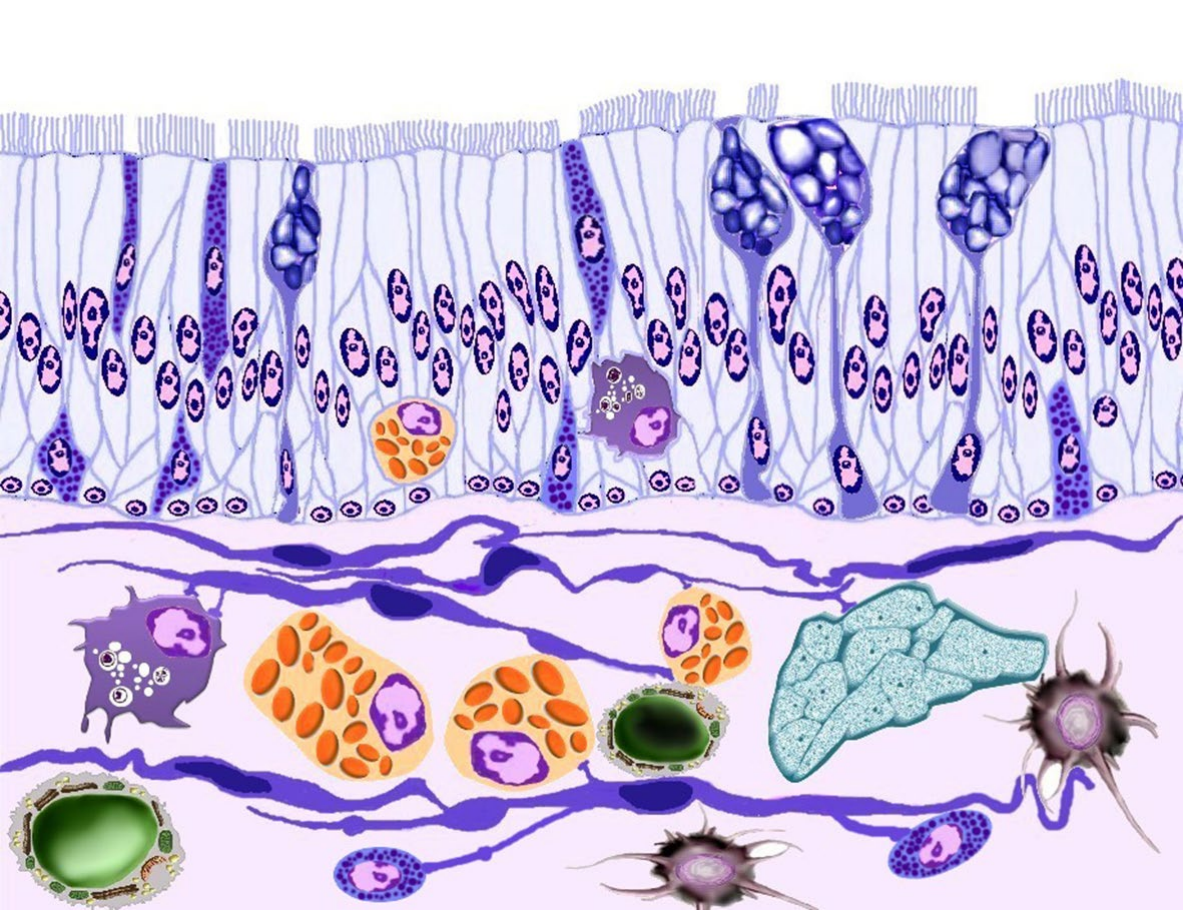
An illustration showed the relation between TCs and other cells in the intestinal blub. TCs established contact with neuroendocrine cells (violet granular cells), nerve fibers (green colored), masts cells (orange colored), dendritic cells (brown colored), lymphocytes (green colored), macrophages (violet colored).

The relation of TCs with endocrine and immune cells was summarized in the illustration figure (Fig. 15).

## Discussion

The current study investigated the distribution, morphology, immunohistochemical characterization of TCs in the intestinal bulb of Grass carp.

We distinguished TCs using a light microscope that had typical morphological features that had a cell body contained the nucleus and telopodes. TCs affinity for nervous tissue-specific staining (methylene blue, Marsland silver stain , silver impregnation, and Giemsa), collagen fiber-specific stains (Crossmon trichrome, Mallory triple, Weigert Van Gieson), polysaccharides-specific stain (PAS,AB pH2.5/PAS) and protein stain (the bromophenol blue) is discussed by Ref.^8^. Methylene blue, Marsland silver stain , silver impregnation stained cytoskeletal elements in the TCs. Cytoskeletal inclusions are stained by methylene blue^72^, Marsland silver stain^73^, silver impregnation^74^. TCs exhibited a strong affinity for carbohydrate staining such as PAS and combined PAS/AB, which revealed neutral polysaccharides. Telocytes had the affinity of protein-detecting stains. They stained positive for bromophenol blue and performic acid with Methylene blue. Proteomic profile analysis is performed for lung TCs, which categorized the functional implications of protein, involved in metabolic processes, cellular processes including cell communication, cytokinesis, cellular component movement, cell cycle. Some proteins implicated in developmental processes including anatomical structure, morphogenesis, mesoderm development, system development, and ectoderm development. Other proteins are linked to *localization* processes such as vesicle mediated transport, protein transport, and ion transport^3^. TCs had strong staining affinity for Sudan black and osmic acid. This may regard to the lipid components of the TCs cell membrane.

In the current study, TCs were located under the epithelium as an individual cell or formed TCs in the lamina propria, between muscle fibers, between muscle bundles, around the myenteric plexus and in the fibrous tissue. The distribution of TCs in the intestinal bulb of the Grass carp was similar to other tubular organs in mammals. In the bovine uterine tubes, TCs are located under the epithelium forming a subepithelial sheath as well as three other sheath; an outer perimuscular, inner perimuscular and intramuscular sheath. TCs are also distributed in the lamina propria, between the SMF and in the serosa^8^. In chicken ileum, ICC subtypes are mentioned according to location. ICC-MY surrounds the myenteric ganglia. ICC-DMP organized in the deep muscular plexus parallel to the circular muscle bundles. ICC-LP is located in the lamina propria^75^. In murine’s GIT, ICC surround the myenteric plexus (Auerbach's plexus) and these are called interstitial cells of Cajal of the myenteric plexus (ICC-MY or ICC-MP) or interstitial cells of Cajal of the Auerbach's plexus [ICC-AP]). Interstitial cells of Cajal of connective tissue septa (ICC-SEP) occur in the connective tissue septa. Intramuscular interstitial cells of Cajal (ICC-IM) are interstitial cells of Cajal of the circular muscle (ICC-CM) and interstitial cells of Cajal of the longitudinal muscle (ICC-LM). Interstitial cells of Cajal of the deep muscular plexus (ICC-DMP) locate in the deep muscular plexus. Interstitial cells of Cajal of the submucosa (ICC-SM) and interstitial cells of Cajal of the submucosal plexus (ICC-SMP) occur in the submucosa and submucosal plexus, respectively. ICC of the subserosa locates in the subserosa^76,77^.

CD-34 is frequently used as a marker for TCs in mammalian and fish species^17,27^. CD34 is a transmembrane phosphoglycoprotein that commonly identified in hematopoietic stem cells and is detected in other progenitor cells such as interstitial cell progenitors, muscle satellite cells, epithelial progenitors, corneal keratocytes, and vascular endothelial progenitors^78^.

TCs expressed chromogranin A that is a highly acidic secretory glycoprotein and is expressed by most neuroendocrine cells. Chromogranin A is closely associated and packed with neurotransmitter peptides and monoamines in secretory granules or synaptic vesicles^79^. Chromogranin A involved in the initiation and regulation of biogenesis of secretory granules and sequestration of hormones in neuroendocrine cells^80^.

TCs established direct contact with different types of immune cells in the intestinal blub of the Grass carp. They were connected to mast cells, dendritic cells, and lymphocytes indicating a contribution in the immune response of the intestinal bulb. TCs–immune cells contact occurs as uniform or multicontact synapses that resemble juxtacrine cell-to-cell signaling sites or chemical synapses. Different types of immune cells are mentioned in contact with TCs, such as lymphocytes, plasma cells, eosinophils, basophils, macrophages, and mast cells^8,27,81^. Moreover, in vitro studies support TCs role in the regulation of immune response via the paracrine pathway. Uterine TCs have a major role in the activation of peritoneal macrophages. Mouse peritoneal macrophages acquired abundant pseudopodia and cytoplasmic secretory granules when co-cultured with TCs. Macrophages increase the section of several cytokines including TNF-α, IL1-R1, and IL-10, but not TGF-β1, IL-1β, IL-23α, and IL-18, suggesting potential role in immunoregulatory and immunosurveillance mechanism^82^. TCs implicated to triggers tissue changes in dendritic-linked immune disease^83^.

C-kit or CD117 is strong markers of stem cells^84,85^. CD117 interacts with the ligand stem cell factor to stimulate cellular differentiation, proliferation, chemotaxis, cell adhesion, and apoptosis^86^. Telocytes are commonly express c-kit. c-kit is a member of the transmembrane kinase receptor. c-kit plays a critical role in TCs functions^87^. C-kit tyrosine kinase is involved in the transduction of intracellular signaling^88^. Telocytes are categorized depending on c-kit immunoaffinity. Some TCs were CD34-positive/c-kit-negative in the human bladder^89^.

TCs had high affinity for immunostaining of cytoskeletal components, particularly the intermediate filaments desmin, and S-100 protein. The term S-10 protein is used based on the physical properties of the protein that is soluble in a 100% saturated solution of ammonium sulfate at neutral pH^90^. S-100 proteins are a member of Ca^+2^-binding proteins. They function as Ca^+2^sensor proteins and promote Ca^+2^signal transduction. S-100 proteins have intracellular and extracellular functions. They combine into specific proteins in cells, e.g., desmin, tubulin, and others^91^. S-100 proteins bind to different types of cell receptors^92,93^. S-100 proteins are implicated in the regulation of cell dynamics and shape through the interaction of cytoskeletal proteins including microfilaments, microtubules, and intermediate filaments^94,95^. They interact with proteins that act as protein Players that regulate the secretory pathway^93^. Thus, S-100 proteins may participate in the regulation of the TC morphology and the secretory functions of TCS. TCS express desmin in the intestinal bulb of the Grass carp. Desmin is a member of the intermediate filament. As the other cytoskeletal elements, desmin has a supportive function to the cells^96^. TCs express desmin in the uterine tube of bovine^8^.

## Conclusion

The current study presented the relation of TCs with different types of immune cells in the intestinal bulb of the Grass carp. We suggest that TCs may have an essential role to maintain intestinal immunity. TCs share histochemical and immunological features of endocrine cells. This may reveal that TCS had endocrine properties. Further studies should investigate TCs proteins profile linked to endocrine function.

## Supplementary information

Supplementary information.

## Data Availability

All data generated or analyzed during this study are included in this published article and its Supplementary Information files.
