# Author Correction: Morphological and immunohistochemical phenotype of TCs in the intestinal bulb of Grass carp and their potential role in intestinal immunity

**DOI:** 10.1038/s41598-021-90399-w

**Published:** 2021-05-31

**Authors:** Hanan H. Abd-Elhafeez, Alaa S. Abou-Elhamd, Soha A. Soliman

**Affiliations:** 1 Department of Anatomy, Embryology and Histology, Faculty of Veterinary Medicine, Assiut University, Assiut, 71526 Egypt; 2 Department of Histology, Faculty of Veterinary Medicine, South Valley University, Qena, 83523 Egypt

Correction to: *Scientific Reports* https://doi.org/10.1038/s41598-020-70032-y, published online 20 August 2020

This Article contained errors.

The version of this Article previously published quoted an incorrect email address for Hanan H. Abd-Elhafeez. Correspondence and requests for materials should be addressed to hhnnzz91@aun.edu.eg

Furthermore, the legend of Figure 14,

“Scanned muscular TCs. (**A**) scanned sample of the intestinal blub. Note the epithelium (violet color, ep), lamina propria (lp, telocytes (blue color), muscle (m, reddish brown color). (**B**, **C**) TCs network (blue colored) in the lamina propria. Note connected teloped (pink colored). (**D**–**F**) TCs (blue colored) between muscle bundles (red colored). (**G**, **I**) TC network between muscle fibers (reddish brown colored). Note serosa (violet colored). (**H**) TCs in serosa.”

now reads:

“Scanned muscular TCs. (**A**) scanned sample of the intestinal blub. Note the epithelium (ep), lamina propria (lp, telocytes (blue color), muscle (m, reddish brown color). (**B**, **C**) TCs network (blue colored) in the lamina propria. Note connected teloped (pink colored). (**D**–**F**) TCs (blue colored) between muscle bundles (red colored). (**G**, **I**) TC network between muscle fibers (brown colored). Note connetced teloped (pink colored). (**H**) TCs (blue color) in serosa.”

Lastly, in the Conclusion section,

“The current study presented the relation of TCs with different types of immune cells in the intestinal bulb of the Grass carp [17].”

now reads:

“The current study presented the relation of TCs with different types of immune cells in the intestinal bulb of the Grass carp.”

These errors have now been corrected in the PDF and HTML versions of the Article.

