# Supplementary material for: Morphological and immunohistochemical phenotype of TCs in the intestinal bulb of Grass carp and their potential role in intestinal immunity
Source: Sci Rep. 2020 Aug 20;10:14039. doi: 10.1038/s41598-020-70032-y (PMC7441181; doi:10.1038/s41598-020-70032-y)

**Morphological and immunohistochemical phenotype of telocytes in intestinal bulb of Grass carpand their potential role in intestinal immunity**

**Hanan H. Abdelhafeez, Alaa S. Abou-Elhamd, Soha A. Soliman**

Fig.1: Negative image of Immunohistochemical staining of the intestinal blub using CD34

immunostained paraffin sections of the intestinal blub for CD34. A: CD34 TCs (double arrowheads) in the subepithelial layer (Ep). B: CD34 TCs (double arrowheads) around the blood vessels (bv).


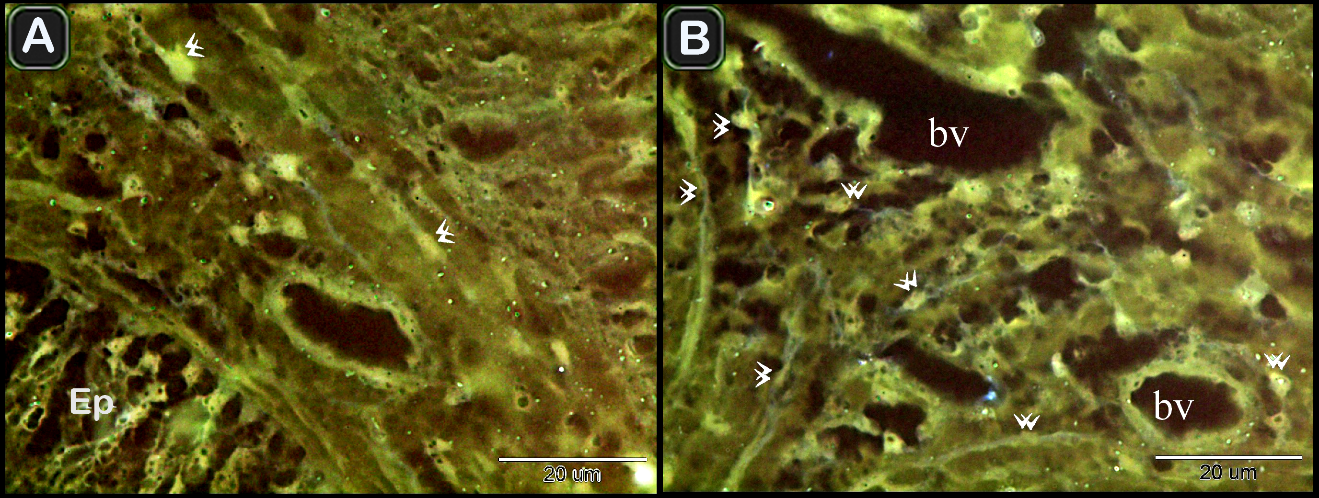


Fig.2: Negative image of immunohistochemical staining of the intestinal blub using CD117

immunostained paraffin sections of the intestinal blub for CD117. A: CD117 positive TCs (arrows) under the epithelium and in the lamina propria. B: CD117 positive sub epithelial TC (arrows). C, E, F: CD117 positive TCs (arrows) in the lamina propria. D: CD117 positive TC (arrows) around the ganglionic cell (g) of the myenteric plexus. G, H I: CD117 positive TC (arrows) within the muscle layers. Note: Telopodes (arrowheads) and smooth muscle fiber (SMF).


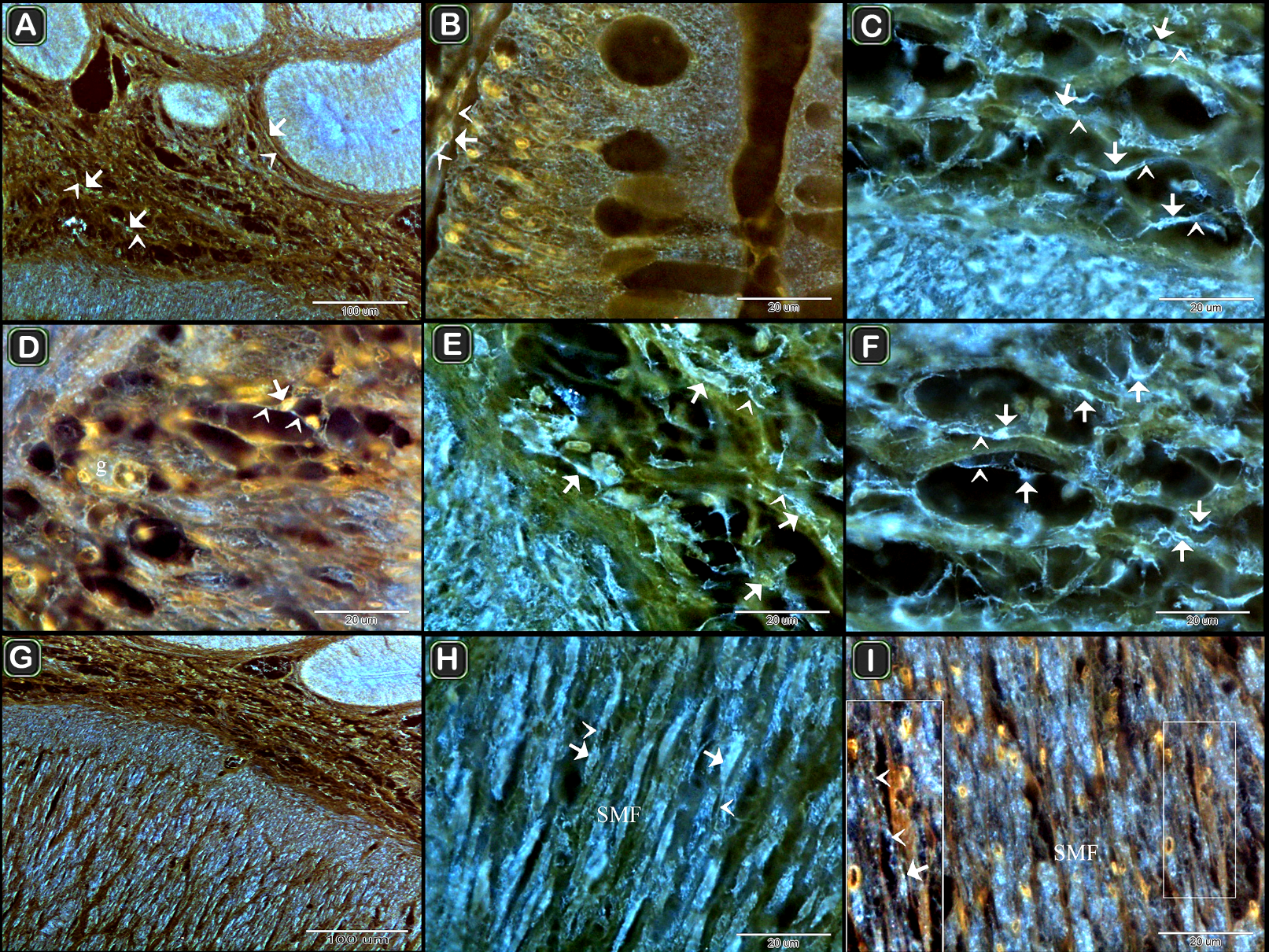


Fig.3: Negative image Immunohistochemical staining of the intestinal blub using desmin and S100-protein. immunostained paraffin sections of the intestinal blub for desmin (A-C) and S100-protein (D-F). A: desmin positive subepithiel TCS (arrows). B: desmin positive TCs (arrows) in the lamina propria. C: desmin positive TCs (arrows) between the muscle bundles. D: S100-protein positive TCs (arrows) under the epithelium. E: S100-protein positive TC (arrows) between the muscle bundles. F: S100-protein positive TC (arrows) connected with nerve fiber (NF). Note: Telopodes (arrowheads)


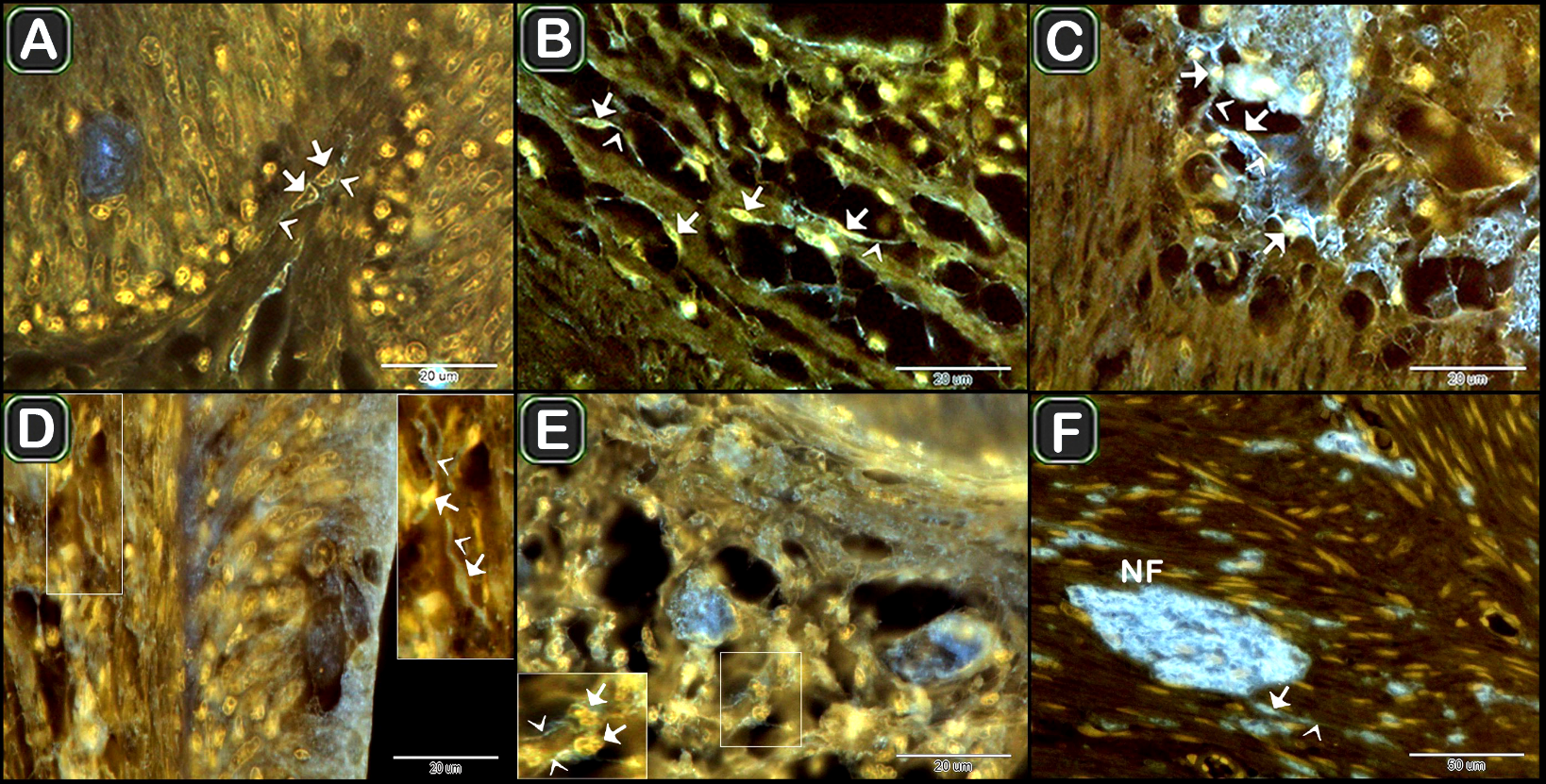


Fig.4:Images of negative control of immunohistochemical staining of the intestinal blub using CD34(A), CD117 (B), Desmin (C), S100 protein (D).


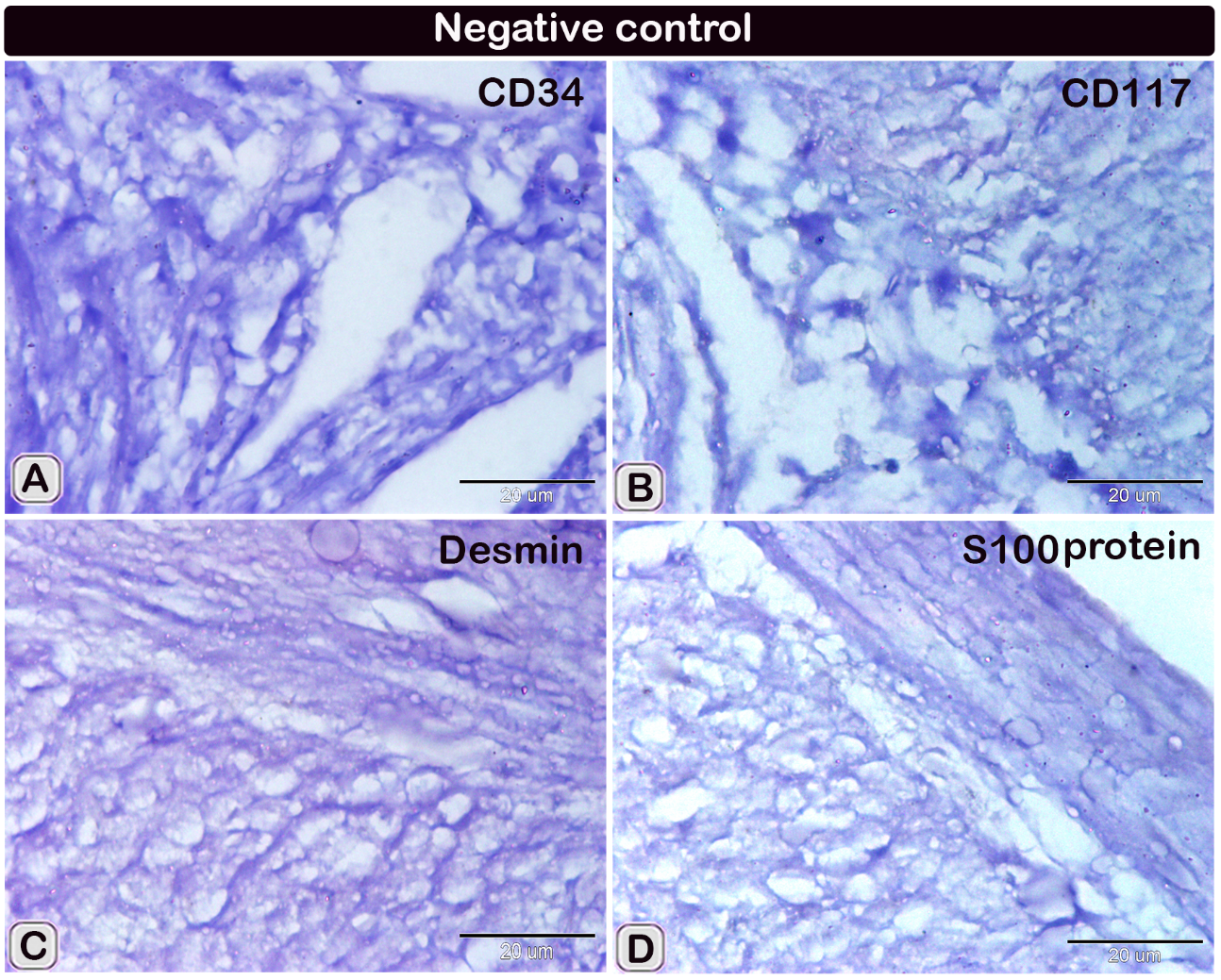


Fig.5: negative images of negative control of immunohistochemical staining of the intestinal blub using CD34(A), CD117 (B), Desmin (C), S100 protein (D).


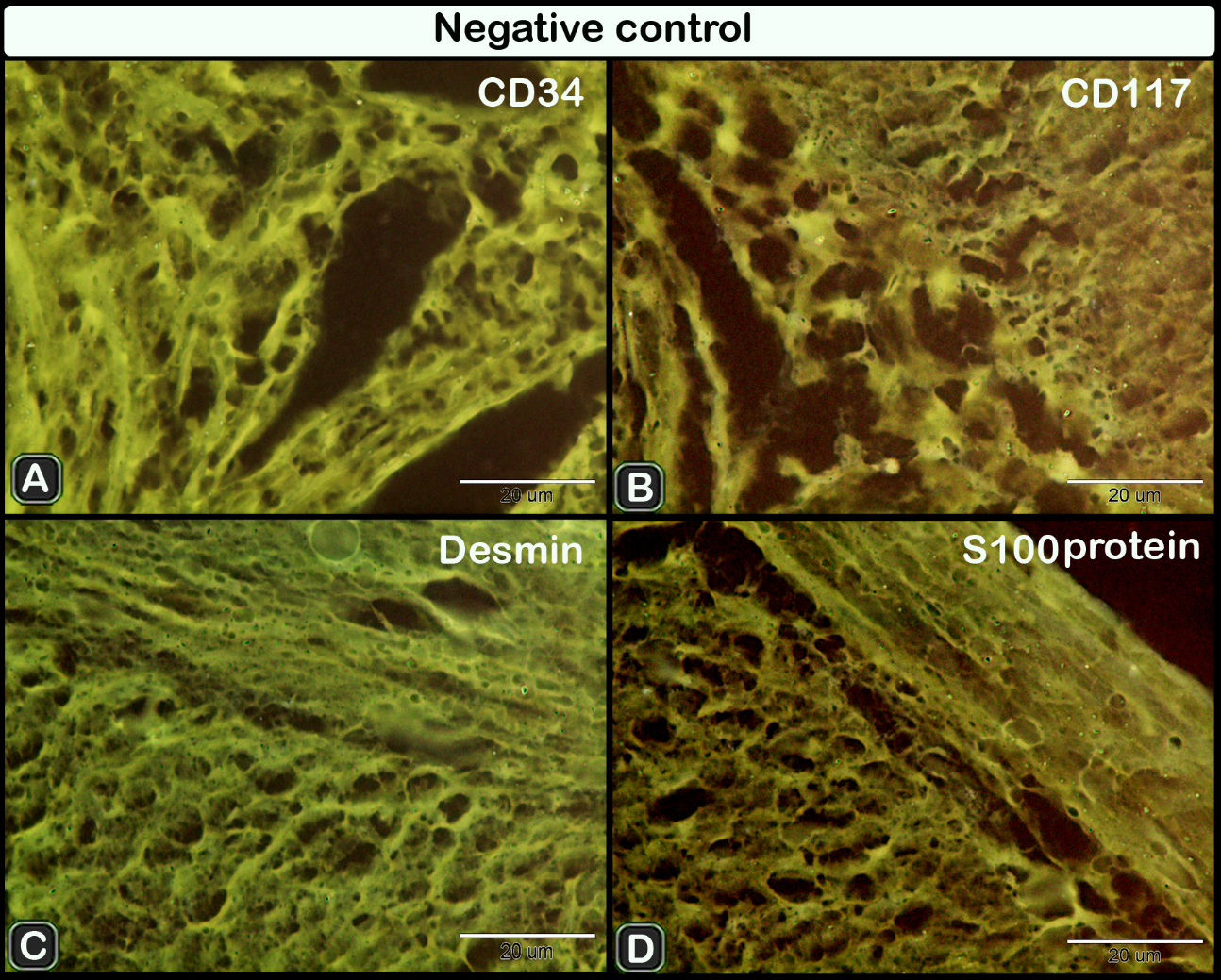


Fig. 6: Negative image of Relation between TCs and epithelial and interstitial endocrine cells using Grimelius’s silver nitrate stain, performic acid with Alcian blue, Marsland stain and immunohistochemical staining using chromogranin A.

Paraffin sections stained by Grimelius’s silver nitrate stain, (A-C), performic acid with Methylene blue (D), Marsland stain (G, H), chromogranin A (E, F, I). A-C: subepithelial TCs (arrows) were closely related to endocrine cells (arrowheads). D: subepithelial TCs (arrows) were closely related to endocrine cells (arrowheads). E: chromogranin positive within the cells of epithelial lining (arrows). F-I: subepithelial and interstitial TCs (arrows) were closely related to endocrine cells (arrowheads).


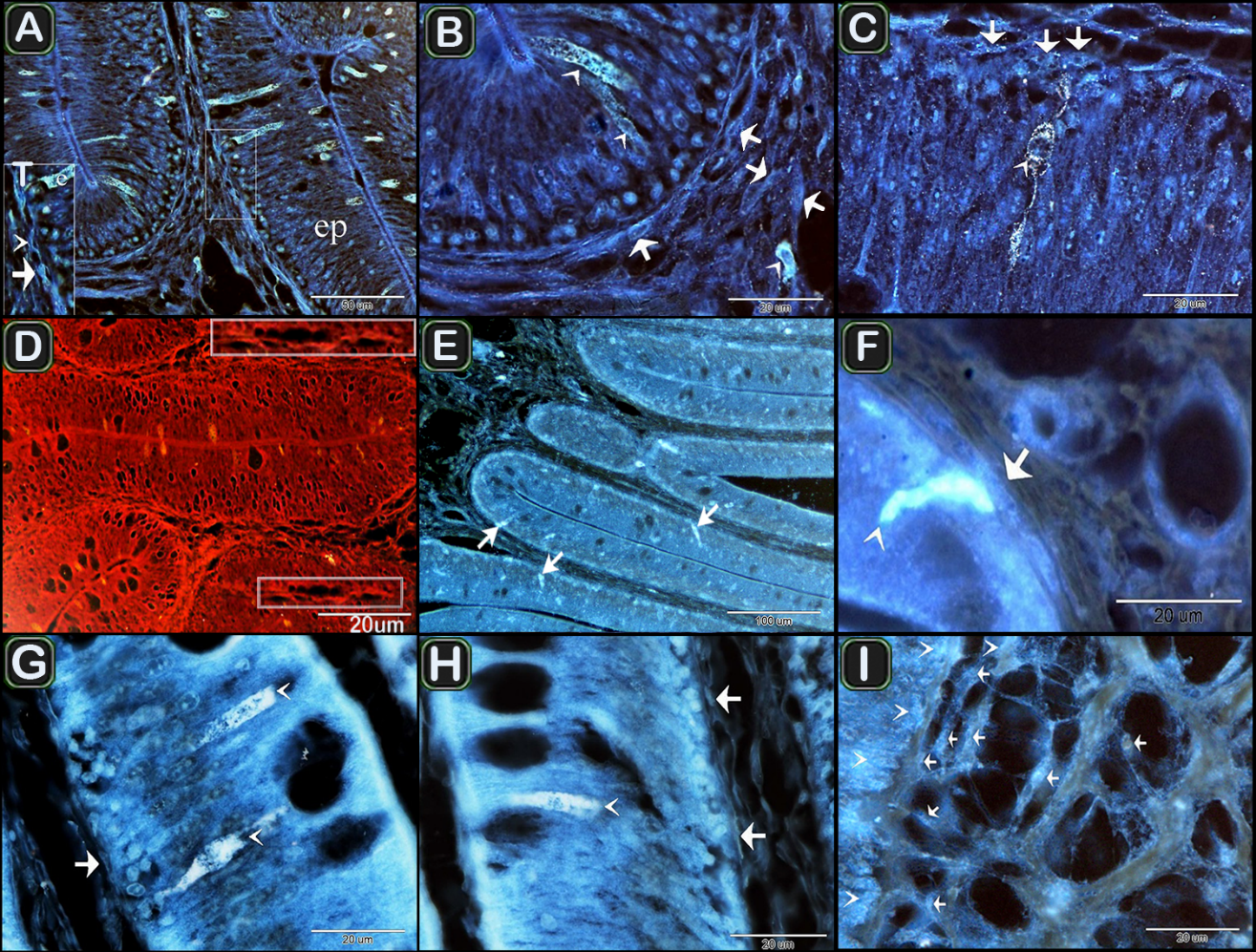


Fig.7: negative control and negative image of immunohistochemical staining of the intestinal blub using chromogranin A.


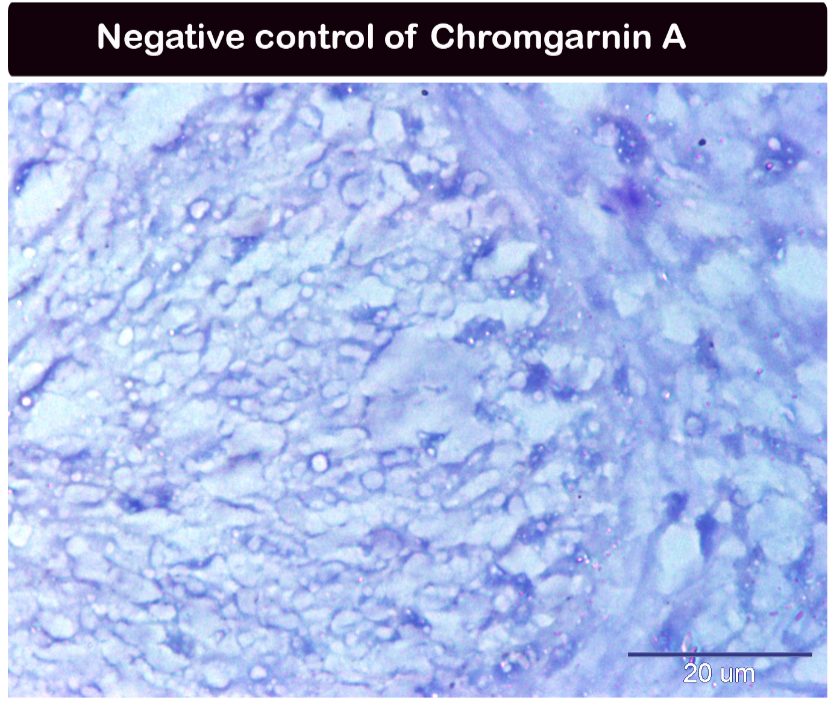


Fig. 8: negative images of negative control of immunohistochemical staining of the intestinal blub using chromogranin A.


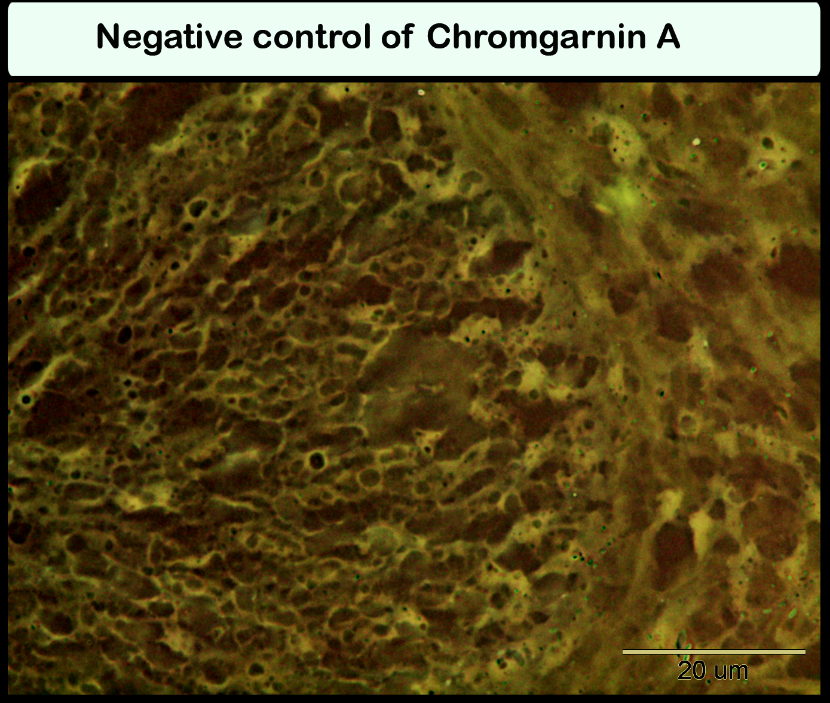

Supplement: Supplementary file 1 — Supplementary information. [file 41598_2020_70032_MOESM1_ESM.docx]
